# Microbial levan potentiates hepatic retention and antitumor activity of a PEGylated benzimidazole–curcumin nanocomplex through TLR2–*FXR*/*FGF15*-associated immunometabolic remodeling in experimental liver cancer

**DOI:** 10.1038/s41598-026-64576-8

**Published:** 2026-08-12

**Authors:** Heba Shawky, Noha E. Ibrahim, Mai N. Amer, Aisha A. K. Al-Ashmawy, Olfat A. Hammam, Dalia B. Fayed, Amany S. Maghraby

**Affiliations:** 1 Therapeutic Chemistry Department, Pharmaceutical and Drug Industries Research Institute, National Research Centre, Dokki, Cairo, 12622 Egypt; 2 Microbial Biotechnology Department, Biotechnology Research Institute, National Research Centre, Dokki, Cairo, 12622 Egypt; 3 Chemistry of Natural and Microbial Products Department, Pharmaceutical and Drug Industries Research Institute, National Research Centre, Dokki, Cairo, 12622 Egypt; 4 Pathology Department, Theodor Bilharz Research Institute, Giza, Egypt

**Keywords:** Nanomedicine, Bacterial levan, Benzimidazole, Curcumin, Host-response modulation, Immunometabolic reprogramming, FXR/FGF15 axis, Liver cancer, Biochemistry, Biotechnology, Cancer, Drug discovery, Immunology

## Abstract

Despite major advances in nanomedicine, therapeutic efficacy remains constrained by unfavorable host responses that influence nanotherapeutic retention, biodistribution, and activity. Biological strategies capable of enhancing nanomedicine performance through modulation of host-response pathways, rather than additional nanoparticle engineering, remain largely unexplored. Herein, we investigated whether a microbial levan produced by *Weissella paramesenteroides* could potentiate the therapeutic activity of a PEGylated β-cyclodextrin-capped benzimidazole–curcumin nanocomplex (BMPE–Cur) in an Ehrlich ascites carcinoma (EAC)-induced hepatic tumor model. Pharmacokinetic, biochemical, histopathological, angiogenic, immunological, molecular, and computational analyses were performed to evaluate therapeutic efficacy and explore underlying mechanisms. The co-administration of levan increased hepatic BMPE accumulation by 2.3-fold and significantly enhanced the therapeutic efficacy of BMPE–Cur, improving survival to 95.45% compared with 60.66% mortality in untreated tumor-bearing mice. Combination therapy produced 86% inhibition of angiogenesis, markedly suppressed tumor-associated biomarkers, oxidative stress, inflammatory mediators, and immunosuppressive cell populations, and restored hepatic function. These effects were accompanied by reduced expression of components of the TLR2/*MyD88*/*NF-κB* pathway, including an 80.05% reduction in *MyD88* and a 78.93% reduction in *NF-κB*, together with 6.59-fold and 7.45-fold increases in *FXR* and *FGF15* expression, respectively. Molecular docking supported potential interactions of BMPE, curcumin, and levan-derived structural motifs with TLR2, while isobolographic analysis demonstrated predominantly synergistic interactions across multiple biological endpoints. These findings support the potential of microbial levan as a promising biological potentiator of nanotherapeutic efficacy, with concurrent enhancement of hepatic nanocomplex retention and coordinated immunometabolic remodeling. More broadly, microbial exopolysaccharides may represent a promising class of host-response modulators, supporting a therapeutic paradigm in which optimizing the biological environment may complement conventional nanocarrier engineering to improve the performance of cancer nanomedicine.

**Supplementary Information:**

The online version contains supplementary material available at 10.1038/s41598-026-64576-8.

## Introduction

Liver cancer remains one of the most aggressive malignancies worldwide and is associated with poor prognosis and limited therapeutic responsiveness, accompanied by profound alterations in redox balance, immune regulation, and metabolic activity^1^. Despite tremendous advances in targeted and immune-based therapies, treatment options remain constrained by therapeutic resistance, dose-limiting toxicities, and disease heterogeneity, while curative interventions such as surgical resection and liver transplantation remain applicable only to a limited proportion of patients^2^. These challenges underscore the need for multimodal therapeutic strategies capable of simultaneously targeting oxidative, inflammatory, immune, and metabolic pathways involved in tumor progression.

Polymeric nanomedicines have emerged as promising platforms for improving the solubility, stability, biodistribution, and therapeutic efficacy of anticancer agents^3^. Through controlled drug release and improved pharmacokinetic behavior, nanotherapeutic systems can enhance drug exposure while reducing systemic toxicity^4^. In our previous study, we developed a PEGylated β-cyclodextrin-capped benzimidazole–curcumin nanocomplex (BMPE–Cur) that combined the complementary anticancer activities of benzimidazole and curcumin within a pH-responsive nano-delivery platform^5^. The nanocomplex demonstrated sustained drug release, potent antiproliferative activity against HepG2 cells, and favorable pharmacokinetic characteristics in healthy animals, supporting its potential as a nanotherapeutic platform for liver cancer^5,6^.

However, whether modulation of host biological responses could further influence the in vivo disposition and therapeutic performance of BMPE–Cur under pathological conditions remained unknown. This challenge is particularly relevant in metabolically active organs such as the liver, where hepatic clearance mechanisms, inflammatory signaling, oxidative stress, and immune dysregulation can substantially affect nanocarrier disposition, tissue retention, and therapeutic responsiveness, thereby limiting treatment efficacy^7,8^. Consequently, improving nanotherapeutic outcomes may require not only optimization of carrier design but also strategies capable of favorably modulating the host biological responses. In this context, increasing evidence suggests that biologically active macromolecules can influence therapeutic responsiveness by modulating host immune, inflammatory, and metabolic pathways^9^.

Among these, microbial exopolysaccharides have attracted growing attention owing to their ability to regulate oxidative homeostasis, inflammatory signaling, and innate immune responses^10,11^. Levan, a β-(2→6)-linked fructan synthesized by several bacterial species, has been associated with antioxidant, immunomodulatory, anti-inflammatory, and antitumor activities in diverse experimental settings^12,13^. These biological properties suggest that microbial levans may function as adjunct modulators capable of influencing nanotherapeutic disposition and efficacy through favorable remodeling of the host biological environment. Consistent with this concept, our previous studies demonstrated the capacity of lactic acid bacteria-derived levans to attenuate inflammatory and oxidative pathology while modulating disease-associated molecular responses in multiple experimental models^14–16^. Nevertheless, whether bacterial levans can enhance nanotherapeutic performance by modulating host biological responses that influence nanocomplex disposition and antitumor efficacy remains largely unexplored.

One plausible biological framework through which microbial polysaccharides may influence therapeutic responsiveness involves the gut–liver axis, a bidirectional communication network linking intestinal microbial signals with hepatic immune and metabolic homeostasis^17^. Within this biological framework, Toll-like receptor 2 (TLR2), a key component of the innate immune system, recognizes pathogen-associated and damage-associated molecular patterns, thereby initiating immune responses^18^. In the liver, activation of TLR2 triggers the MyD88/NF-κB signaling cascade, promoting inflammatory cytokine production, angiogenesis, and tumor-supportive immune responses that contribute to hepatic carcinogenesis and disease progression^19^. Beyond immune regulation, the gut–liver axis also encompasses metabolic signaling pathways that govern hepatic homeostasis and xenobiotic metabolism^20^. A key component of this axis is the farnesoid X receptor (FXR) and its downstream effector, fibroblast growth factor 15 (FGF15), which together form a central regulatory pathway linking intestinal and hepatic physiology via bile acid–mediated signaling^21^. In addition to maintaining metabolic homeostasis, FXR/FGF15 signaling regulates hepatic inflammatory responses, drug-metabolizing enzymes, and transporter systems involved in xenobiotic disposition^22,23^.

Given that dysregulation of FXR/FGF15 signaling has been associated with liver injury, metabolic dysfunction, and hepatocarcinogenesis, whereas restoration of this pathway has been linked to improved hepatic function and therapeutic responsiveness^24,25^, the modulation of this pathway may influence the pharmacokinetic behavior and hepatic retention of therapeutic agents.

Based on these insights, we sought to determine whether a *Weissella paramesenteroides*-derived levan could function as a biological potentiator of nanotherapeutic efficacy in experimental liver cancer. Given the established roles of the TLR2/MyD88/NF-κB pathway in tumor-associated inflammatory activation and the FXR/FGF15 pathway in hepatic metabolic homeostasis and xenobiotic disposition, these complementary signaling axes were investigated as candidate mechanisms through which microbial levan might influence nanotherapeutic performance. To test this hypothesis, we evaluated the effects of levan co-administration on the pharmacokinetic behavior, therapeutic efficacy, and mechanistic determinants of BMPE–Cur activity in an EAC-induced hepatic tumor model. Although this model does not fully recapitulate the molecular heterogeneity of human hepatocellular carcinoma, it reproduces several hallmarks of tumor-associated hepatic pathology, including angiogenesis, oxidative stress, inflammatory activation, and immune dysregulation^26^. These pathological features provide a biologically relevant context for investigating host-response pathways, including TLR2-associated inflammatory signaling. Through integrated biodistribution, biochemical, histopathological, immunological, and molecular analyses, we investigated whether microbial levan-mediated host modulation is associated with enhanced hepatic nanocomplex retention and antitumor efficacy without inducing additional hepatotoxicity.

## Materials and methods

### Chemicals, reagents, and drug synthesis

The parent benzimidazole compound, 1-(6-(1 H-benzo[d]imidazol-2-yl)−2-methylpyridin-3-yl) ethanone (BMPE), was synthesized as previously reported by Abdel-Karim et al.^27^, whereas the polymeric benzimidazole–curcumin nanocomplex (BMPE–Cur) was prepared according to our optimized protocol^5^. Unless otherwise stated, all chemicals and analytical-grade solvents were purchased from Sigma-Aldrich (USA).

### Production and purification of the microbial levan

Levan was produced from *Weissella paramesenteroides* via submerged fermentation^28^. Briefly, 5 L of de Man, Rogosa, and Sharpe (MRS) broth were inoculated with 2% (v/v) actively growing bacterial culture (~ 10⁸ CFU/mL) and incubated under controlled conditions for 24–48 h. Following fermentation, cultures were centrifuged for 20 min at 4 °C to remove bacterial cells. The supernatant was adjusted to pH 6.8 and heat-treated at 100 °C for 30 min to inactivate enzymes. After clarification by centrifugation, levan was precipitated using an equal volume of ice-cold absolute ethanol and incubated at 4 °C for 18 h. The precipitate was collected by centrifugation, washed with distilled water, dialyzed against deionized water at 4 °C for 24 h, lyophilized, and stored at 4 °C until use.

### Structural and physicochemical characterization

The structural features of the bacterial exopolysaccharide were evaluated using Fourier-transform infrared spectroscopy (FT-IR; Jasco 6100, Japan) over 400–4000 cm⁻¹, proton nuclear magnetic resonance (^1H NMR; Bruker 400 MHz, DMSO-d₆), and UV–Vis spectroscopy (T80+, PG Instruments, UK; 190–800 nm). These analyses were performed to confirm functional group composition and structural integrity before in vivo studies. The physicochemical characteristics of BMPE–Cur, including its average particle size (467.7 nm) and zeta potential (− 28.24 mV), as well as its pharmacokinetic profile in healthy animals, have been reported previously^5,6^. Therefore, the present study focused on evaluating the influence of microbial levan on the therapeutic performance and endpoint hepatic retention of BMPE–Cur in a pathological tumor model rather than re-characterizing the nanocomplex.

### Animal model and housing conditions

Seventy female Swiss albino mice (22–25 g; 8–10 weeks old) were procured from the Animal House of the National Research Centre (NRC, Cairo, Egypt). The animals were housed in polypropylene cages under standardized environmental conditions, including a 12 h light/dark cycle, controlled ambient temperature (25 ± 2 °C), and relative humidity of 30–60%, within a well-ventilated facility. Standard laboratory chow and water were provided ad libitum. Before the initiation of experimental procedures, all animals were acclimatized for one week. In compliance with the ARRIVE Guidelines 2.0, animal welfare was stringently monitored throughout the study. Health assessments were performed twice daily by a blinded veterinarian at the NRC Animal House, and clinical observations included monitoring for signs of pain or distress, fatigue, erythema of the skin or fur, abnormal ocular discharge, and mortality. Behavioral and neurological parameters, such as ataxia, depressive-like manifestations, hypoactivity or hyperactivity, and changes in locomotor function, were systematically documented to enable early detection of potential adverse effects.

### Tumor induction

Ehrlich ascites carcinoma (EAC) cells were obtained from the National Cancer Institute, Cairo University (Cairo, Egypt). Ascitic tumor cells were maintained via serial intraperitoneal (i.p.) passage in female Swiss albino mice as previously described^29^. For tumor induction, viable EAC cells (5.0 × 10⁶ cells/mouse in 250 µL PBS) were injected i.p according to the method described by Dos Santos et al.^25^, with minor modifications.

### Dose selection and treatment regimen

Seven to ten days post-inoculation, mice exhibiting stable tumor development were randomly assigned to experimental groups (Table 1). Dose selection was based on previously reported safety and pharmacological data. BMPE–Cur (5 mg/kg/day) was selected within its established safe range^27^, and the parent BMPE compound was administered at a dose of 0.13 mg/kg/day to match its equivalent loading within the nanocomplex^5^, thereby enabling a direct comparison between the free drug and nanoformulated counterpart. Doxorubicin (DOX; 10 mg/kg/week) was used as a reference chemotherapeutic agent following established protocols^30^. For the bacterial levan, dose selection was guided by preliminary tolerability and feasibility assessments, given that this study represents its first in vivo evaluation. Initial dose-ranging experiments investigated daily levan doses between 0.05 and 1 mg/kg to define a tolerable exposure window. A dose of 100 µg/kg/day was selected as the highest well-tolerated dose for repeated administration over 15 consecutive days. During preliminary dose-optimization experiments (0.05–1 mg/kg/day), higher levan doses appeared to enhance the therapeutic effects of BMPE–Cur but were also associated with reduced survival. Therefore, 100 µg/kg/day was selected as the optimal dose providing the best balance between efficacy and tolerability (pilot data not shown). Different treatments were administered for 15 consecutive days according to previously established protocols^31^. At the end of the experimental period, body weight and abdominal circumference were recorded before sample collection.

**Table 1:** Experimental groups and treatment regimens (*n* = 12 mice/group).

Group	Intervention
Sham (Vehicle control)	Vehicle only, equivalent dose of physiological saline, oral (o.p.)
Ehrlich ascites carcinoma (EAC) (untreated control)	5.0 × 10⁶ of EAC cells, intraperitoneal (i.p.), no treatment
Parent benzimidazole compound (BMPE)	0.13 mg/kg/day, o.p.
Benzimidazole-Curcumin nanocomplex (BMPE-Cur)	5 mg/kg/day, o.p.
Combination therapy	5 mg/kg/day of BMPE-Cur NPs + 100 µg/kg/day levan, o.p.
Reference drug	10 mg/kg of Doxorubicin (2 mg/mL), i.p., once weekly
Levan only	100 µg/kg/day of purified levan, o.p.

### Sampling

After the completion of the treatment protocol, blood samples were collected from the tail vein into heparinized tubes, and animals were euthanized via intraperitoneal administration of pentobarbital (100 mg/kg, i.p.). The collected blood was divided into two equal portions. One portion was centrifuged at 3000 ×g for 10 min at room temperature to obtain plasma, which was subsequently stored at − 20 °C until further analysis. The second portion was used for the isolation of peripheral blood mononuclear cells (PBMCs) by density gradient centrifugation using Ficoll^®^ Paque Plus (Sigma-Aldrich, USA), in accordance with the manufacturer’s protocol. Liver tissues were excised, rinsed with phosphate-buffered saline (PBS), and subdivided into three portions. The first portion was immediately snap-frozen and stored at − 80 °C for molecular analyses. The second portion was fixed in 10% formaldehyde prepared in PBS for histopathological evaluation. The third portion was homogenized in ice-cold PBS (pH 7.4) to prepare 10% (w/v) tissue homogenates. These homogenates were centrifuged at 5000 ×g for 5 min at 4 °C, and the resulting supernatants were collected for subsequent biochemical and immunological assays. For chromatographic quantification of BMPE and curcumin in liver tissue, an aliquot of the homogenate supernatant was mixed with acetonitrile at a ratio of 4:1 (v/v) and centrifuged at 5000 ×g for 15 min at room temperature. The clarified supernatants were then subjected to LC–MS analysis.

### Pharmacokinetic and hepatic biodistribution analysis

The pharmacokinetic profile and hepatic distribution of the nanocomplex were assessed using liquid chromatography–mass spectrometry (LC–MS). Quantitative determination of parent (unmetabolized) BMPE and curcumin in liver tissues was performed by liquid chromatography–electrospray ionization–tandem mass spectrometry (LC–ESI–MS/MS). Chromatographic separation was conducted using an ExionLC AC system, coupled to a SCIEX Triple Quad 5500 + MS/MS instrument equipped with an electrospray ionization (ESI) source (AB SCIEX, Redwood City, CA, USA)^6^. Milli-Q water and LC-grade acetonitrile were used throughout the analysis. Separation was achieved on a ZORBAX Eclipse Plus C18 column (4.6 × 100 mm, 1.8 μm; Agilent Technologies, Santa Clara, CA, USA) maintained at 25 °C. The mobile phase consisted of a binary mixture of 0.1% formic acid in water (40%, v/v; solvent A) and acetonitrile (60%, v/v; solvent B), delivered at a flow rate of 0.8 mL/min. The injection volume was 3 µL per sample. As BMPE is an in-house synthesized compound without a commercially available reference standard, calibration curves were generated by spiking blank liver homogenate supernatants with known concentrations (5–500 ng/mL) of purified BMPE and curcumin. The calibration equations obtained were as follows: BMPE, *Y* = 2896.11613*X* + 10.46878 (*r* = 0.99953, *r²* = 0.99906); curcumin, *Y* = 9737.89729*X* − 1771.52541 (*r* = 0.99921, *r²* = 0.99841) (Supplementary Data File 1). Mass spectrometric detection was performed in positive ionization mode under optimized conditions: curtain gas, 25 psi; ion spray voltage, 5500 V; source temperature, 400 °C; and ion source gases 1 and 2 set at 55 psi. Quantification was carried out using multiple reaction monitoring (MRM), with compound-specific transitions and optimized parameters summarized in Supplementary Table 1. Data acquisition and processing were performed using Analyst^®^ software version 1.5 (AB SCIEX, Redwood City, CA, USA).

### In silico metabolic predictions

The chemical structures and canonical MOL files of the principal pharmacophores examined in this study, namely BMPE, curcumin, and fructose (the predominant monosaccharide constituent of levan, ~ 80%^28^, were retrieved from the Cheminfo platform (http://www.cheminfo.org/Chemistry/Name_to_structure/index.html). These structural datasets were subsequently employed to predict Phase I and Phase II metabolic pathways, with particular focus on potential interactions with and inhibition of cytochrome P450 (CYP450) enzymes, using the PreMetabo online prediction tool (https://premetabo.webservice.bmdrc.org)^32^.

### Biochemical assessment of liver functions and redox status

Liver function parameters, including alanine aminotransferase (ALT), aspartate aminotransferase (AST), total and direct bilirubin (BiL), and alkaline phosphatase (AP), were measured in plasma using the corresponding commercial kits from Biodiagnostic (Cairo, Egypt) in accordance with the manufacturer’s instructions. Redox status markers, including nitric oxide (NO), malondialdehyde (MDA), reduced glutathione (GSH), catalase, and superoxide dismutase activities, were assessed in liver homogenates using the corresponding Biodiagnostic (Cairo, Egypt) kits.

### Evaluation of tumor-associated and angiogenic biomarkers

Peritoneal angiogenesis was qualitatively assessed by surgically exposing the peritoneal cavity of each mouse for macroscopic evaluation of vascularization, following the method described by Hajra et al.^33^. Evaluations were performed independently by two investigators blinded to treatment allocation, yielding high inter-rater agreement (Cohen’s κ = 0.903; > 90% reliability). Vascular density was quantified using ImageJ software^26^. To validate the automated analysis, a subset of samples was additionally subjected to manual planimetric assessment, demonstrating strong concordance between the two methods (R² = 0.8375). In parallel, plasma and liver tissue homogenates were analyzed for alpha-fetoprotein (AFP; Cat# SL0035Mo), carcinoembryonic antigen (CEA; Cat# SL0120Mo), and vascular endothelial growth factor receptor (VEGFR; Cat# SL0561Mo) using the corresponding mouse ELISA kits (SunLong Biotech, China), in accordance with the manufacturer’s instructions. Absorbance was measured at 450 nm, and cut-off values were determined based on the minimum detection limits specified for each assay. Detailed performance characteristics, including assay sensitivity, dynamic range, and intra- and inter-assay variability, are provided in the respective manufacturer manuals and can be accessed using the cited catalog numbers.

### Immunological studies

Immunological parameters were quantified in plasma, peripheral blood mononuclear cells (PBMCs), and liver tissue homogenates using commercially available mouse ELISA kits (SunLong Biotech, China), in accordance with the manufacturer’s instructions. The analyzed cytokines and immune markers included interleukin (IL)−6 (Cat# SL0326Mo), tumor necrosis factor (TNF)-α (Cat# SL0547Mo), IL-10 (Cat# SL0310Mo), C-X-C motif chemokine ligand (CXCL)−1 (Cat# SL0916Mo), CD4⁺ T helper (Th) lymphocytes (Cat# SL0716Mo), CD8⁺ cytotoxic T (Tc) lymphocytes (Cat# SL0717Mo), and CD25⁺ regulatory T (Treg) lymphocytes (Cat# SL0875Mo). Hepatic expression of Toll-like receptor 2 (TLR2) was determined using an in-house enzyme-linked immunosorbent assay (ELISA) adapted from the method originally described by Engvall and Perlman^34^ and later modified by Razin et al.^35^, with minor modifications. Briefly, flat-bottom polyvinyl microtiter plates (ALTO, Italy) were coated overnight at 4 °C with 50 µL of liver homogenate supernatants (1 µg protein) diluted in carbonate buffer (pH 9.6). Plates were washed three times with phosphate-buffered saline (PBS) containing 0.05% Tween-20, followed by blocking with 200 µL of 3% bovine serum albumin (BSA) in PBS–0.05% Tween-20 for 1 h at 37 °C. After washing, 50 µL of rabbit polyclonal anti-TLR2 antibody (1:1000; Abcam, UK; Cat. No. ab213676) diluted in PBS–0.05% Tween-20 was added to each well and incubated for 2 h at 37 °C. Bound primary antibodies were detected using 50 µL of horseradish peroxidase (HRP)-conjugated goat anti-rabbit IgG (H&L) (1:10,000; Abcam, UK; Cat# ab205718) prepared in blocking buffer. Color development was achieved by adding 100 µL of 3,3′,5,5′-tetramethylbenzidine (TMB) substrate (Thermo Fisher Scientific, USA; Cat. No. N301) and incubating the plates in the dark at room temperature. The reaction was terminated with 50 µL of 2 N sulfuric acid, and absorbance was measured at 490 nm using a Varioskan™ LUX multimode microplate reader (Thermo Fisher Scientific, USA). The cut-off value for positivity was defined as twice the optical density at 490 nm (OD₄₉₀) of the negative control.

### Molecular analyses

Hepatic mRNA expression levels of myeloid differentiation factor 88 (*MyD88*), nuclear factor kappa B (*NF-κB*), farnesoid X receptor (*FXR*), and fibroblast growth factor 15 (*FGF15*) were quantified in control and treated Ehrlich ascites carcinoma (EAC) mice using quantitative polymerase chain reaction (qPCR). Total RNA was isolated from liver tissues using the RNeasy Kit (Cat#74004; QIAGEN, Germany) in accordance with the manufacturer’s protocol. Quantitative amplification was carried out on a Rotor-Gene^®^ Q real-time PCR system (QIAGEN, Germany) in a final reaction volume of 50 µL. Each reaction contained 1 µL of iScript reverse transcriptase for one-step RT-PCR (Cat. No. 345–0412; Bio-Rad, USA), 25 µL of 2× SYBR^®^ Green RT-PCR reaction mix supplemented with magnesium chloride and 0.4 mM of each deoxynucleotide triphosphate (dATP, dCTP, dGTP, and dTTP), 200 ng of purified RNA template, and 10 µM of each gene-specific primer. The final volume was adjusted with nuclease-free water. Thermal cycling conditions comprised an initial reverse transcription step at 50 °C for 10 min, followed by enzyme activation at 95 °C for 5 min. Amplification was performed over 45 cycles, each consisting of denaturation at 95 °C for 10 s and primer-specific annealing at the designated temperature for 30 s, during which fluorescence signals were acquired. To confirm amplification specificity and exclude primer–dimer formation, a post-amplification melting curve analysis was conducted (95 °C for 1 min, 55 °C for 1 min, followed by incremental increases of 0.5 °C per cycle for 80 cycles). Relative gene expression levels were calculated using the comparative threshold cycle (^ΔΔ^Ct) method and normalized to the geometric mean expression of the housekeeping gene glyceraldehyde-3-phosphate dehydrogenase (GAPDH). Primer amplification efficiency was validated through standard curve analysis based on the linear relationship between Ct values and the logarithm of DNA copy number, assuming an amplification factor of 2. All primer sets exhibited amplification efficiencies ranging from 92.14% to 101.77%, with correlation coefficients (R²) exceeding 0.99. Primer sequences, annealing temperatures, and amplification efficiencies are provided in Supplementary Table 2^14,36–38^.

### Histopathological examination

Formalin-fixed liver tissues were dehydrated in a graded ethanol series (70–100%), cleared in xylene, and embedded in paraffin. Section (5 μm) were mounted on glass slides and stored at room temperature. Before staining, sections were deparaffinized in xylene and rehydrated through descending ethanol concentrations. Hematoxylin and eosin (H&E) staining was performed following Suvarna and Layton^39^. Sections were examined under a light microscope, representative images captured digitally, and histopathological analysis was conducted by investigators blinded to the experimental groups.

### Molecular docking simulation

Molecular docking analysis was performed to explore the potential capacity of nanocomplex and levan-derived constituents (benzimidazole, curcumin, and fructose units) to directly interact with the TLR2 ligand-binding domain. The synthetic phospholipid PE-DTPA (native ligand) and the lipopeptide Pam2CSK4 (TLR2 agonist; PDB ID: 3A7C) were included as reference controls^40^. The crystallographic structure of TLR2 was retrieved from the Protein Data Bank (https://www.rcsb.org/) and subjected to docking using AutoDock Vina implemented in the PyRx platform (https://pyrx.sourceforge.io/)^41^, following established protocols^42,43^. Protein preparation and energy minimization were performed using the YASARA server^44^, while ligand–protein interactions were visualized using BIOVIA Discovery Studio 2024 (https://discover.3ds.com/). Docking reliability was confirmed by re-docking the native ligand into the TLR2 binding pocket, yielding an RMSD of 0.7 Å. Simulations targeted the hydrophobic cavity corresponding to the Pam2CSK4 binding site, a region known to be competitively occluded by staphylococcal superantigen-like proteins (SSLs)^45^. A grid box of 25 × 25 × 25 Å was centered at X = − 25.56, Y = − 7.64, and Z = − 9.68 to encompass the entire ligand-binding domain.

### Mathematical evaluation of BMPE-Cur – levan interactions

The interaction between BMPE–Cur and levan was evaluated via isobolographic analysis to determine whether their combination was additive, synergistic, or antagonistic. The Combination Index (CI) was calculated using CompuSyn software^46^, which applies the median-effect principle to compare the observed combined effect with that expected if the agents acted independently. In this framework, CI = 1 denotes an additive effect, CI < 1 indicates synergism, and CI > 1 indicates antagonism. This approach provides a quantitative assessment of how the two agents modulate each other’s activity.

### Statistical analysis

The sample size was calculated using G*Power version 3.1.9.7. A priori analysis comparing the vehicle control and untreated EAC groups revealed an 11.527-fold increase in serum AFP levels in the untreated group (Fig. S1). To detect this effect with a statistical power of 95% (1 − β) at a significance level of α = 0.05, the minimum required sample size was two mice per group; however, twelve mice were allocated to each group in accordance with Dos Santos et al.^26^. Numerical data were analyzed using GraphPad Prism version 9.0.2 (GraphPad Software, San Diego, CA, USA). Survival was analyzed using the Mantel–Cox (log-rank) test. All other outcome measures were analyzed using the Kruskal–Wallis test followed by Bonferroni-corrected pairwise comparisons following a significant overall test to control the family-wise Type I error rate within each endpoint. Each experimental group included at least six independent animals (biological replicates). For biochemical, immunological, and molecular assays, each sample was measured in technical triplicate, and the mean value for each animal was used for statistical analysis. Data are presented as mean ± SD. Two-tailed P values < 0.05 were considered statistically significant.

## Results

### Structural confirmation of microbial levan

Spectroscopic analyses confirmed that the purified polysaccharide is a levan-type fructan, consistent with previously reported microbial levans (Figs. S2 – S4). The obtained spectral signatures validated both its structural identity and high purity before biological evaluation. Comprehensive spectral characterization of the microbial levan is provided in Supplementary Data File 2.

### Effect of therapeutic interventions on survival rate and EAC-associated morbidity

**Fig. 1:**
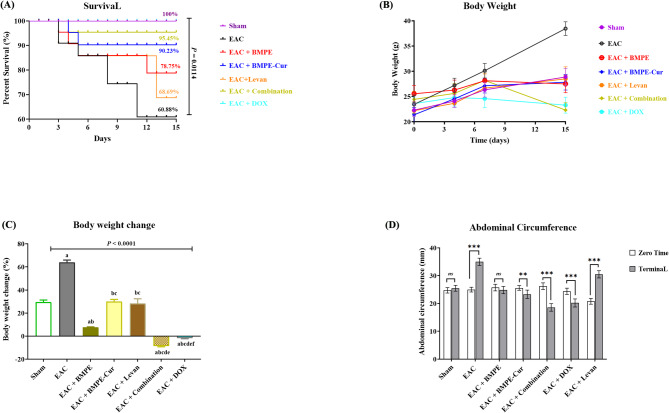
Effects of therapeutic interventions on survival, body weight, and abdominal circumference in EAC-bearing mice. **(A)** Kaplan–Meier survival curves showing improved survival in all treated groups compared with untreated EAC mice. No mortality was observed in the sham or DOX groups (*n* = 12 per group). Terminal survival rates reached 78.57% (parent BMPE, *n* = 8), 90.23% (BMPE–Cur, *n* = 10), 95.45% (BMPE–Cur + levan, *n* = 11), and 68.69% (levan, *n* = 7), compared with 39.34% (*n* = 6) in untreated EAC mice (*P* = 0.0114). **(B**,** C)** Changes in body weight and **(D)** abdominal circumference during the 15-day experimental period. Untreated EAC mice exhibited progressive increases in body weight and abdominal circumference, reaching 63.9% and 40.0% above baseline values, respectively, and 33.18% and 39.21% higher than sham controls (*P* < 0.0001). All treatments significantly reduced body weight and abdominal circumference relative to untreated EAC mice (*P* < 0.0001). Parent BMPE and BMPE–Cur normalized both parameters, whereas DOX and the combination produced moderate reductions relative to sham controls (*P* < 0.0001). Notably, mice receiving BMPE–Cur + levan displayed a lower abdominal circumference than DOX-treated animals (*P* = 0.0178). Data are presented as mean ± SD.

No mortality was observed in the sham or doxorubicin (DOX) groups, whereas the untreated EAC mice exhibited a 60.66% mortality rate by day 15 (Fig. 1A). All treatments significantly improved survival (*P* = 0.0114), reaching 78.57% (parent BMPE), 90.23% (BMPE–Cur), 95.45% (BMPE–Cur + Levan), 100% (DOX), and 68.69% (Levan). Untreated EAC mice developed progressive ascites, evidenced by marked increases in body weight (+ 63.9 ± 2.06%) and abdominal circumference (+ 40.0 ± 4.55%) vs. baseline, and by 33.18 ± 4.83% and 39.21 ± 3.71% relative to sham control (*P* < 0.0001 for all; Fig. 1B – D). All interventions significantly attenuated these parameters from day 7 onward (*P* < 0.0001). By day 15, body weight was reduced by 28.48 ± 3.82% (parent BMPE), 27.62 ± 2.98% (BMPE–Cur), 41.92 ± 3.61% (combination), 39.40 ± 4.85% (DOX), and 25.38 ± 3.16% (Levan), with corresponding decreases in abdominal circumference of 29.06 ± 3.16%, 33.34 ± 2.87%, 46.86 ± 4.11%, 42.29 ± 3.65%, and 12.86 ± 2.28%, respectively, compared to the untreated animals. Parent BMPE normalized both body weight and abdominal circumference relative to sham control, although a modest reduction in body weight change percentage remained evident (*P* < 0.0001). Likewise, BMPE–Cur and levan monotherapies produced terminal body weight changes comparable to those of sham animals. In contrast, DOX and BMPE–Cur + levan induced moderate but significant reductions in terminal body weight (–19.29 ± 3.04% and − 22.65 ± 2.94%, respectively) and abdominal circumference (–20.78 ± 2.74% and − 27.05 ± 3.33%, respectively) relative to sham controls (*P* < 0.0001 for all comparisons). However, whereas DOX-treated mice exhibited no significant difference between baseline and terminal body weight values, the combination treatment produced a significant reduction in body weight relative to baseline (*P* = 0.0214). Notably, despite comparable body weights, mice receiving BMPE–Cur + levan displayed a significantly smaller abdominal circumference than those treated with DOX alone (*P* = 0.0178).

### Effect of therapeutic interventions on tumor-associated hepatic dysfunction

**Fig. 2:**
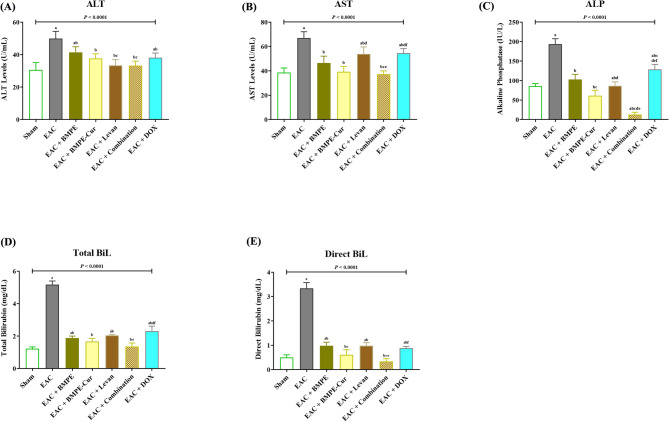
Effect of different treatments on the liver function biomarkers in EAC mice. Untreated EAC mice showed marked elevations in ALT **(A)**, AST **(B)**, alkaline phosphatase **(C)**, total bilirubin **(D)**, and direct bilirubin **(E)** compared with sham control (*P* < 0.0001). All treatments significantly ameliorated these changes (*P* = 0.01 – < 0.0001). Parent BMPE and BMPE–Cur normalized ALT, AST, and AP, with BMPE–Cur additionally normalizing direct bilirubin. Combination therapy restored all liver parameters to sham levels and reduced AP below sham (*P* < 0.0001). The levan monotherapy normalized ALT and AP but showed higher AST and direct bilirubin than BMPE–Cur or combination therapy. DOX showed partial improvement but was less effective than BMPE–Cur or combination treatment. Data are mean ± SD. Statistical annotations: “a” vs. sham; “b” vs. EAC control; “c” vs. parent BMPE; “d” vs. BMPE–Cur; “e” vs. levan; “f” vs. combination.

Biochemically, the untreated EAC mice displayed pronounced hepatic injury, with ALT, AST, and ALP elevated by 38.39 ± 5.34%, 42.20 ± 4.53%, and 55.29 ± 3.42%, respectively (Fig. 2A–C), concurrent with 5 ± 0.93-fold and 4.69 ± 0.45-fold increases in total and direct bilirubin (*P* < 0.0001; Fig. 2D, E). All interventions significantly reduced ALT (–16.90% to − 33.33%), AST (–18.46% to − 44.32%), ALP (–33.37% to − 93.26%), total bilirubin (–62.30% to − 77.87%), and direct bilirubin (–58.56% to − 86.90%) (*P* < 0.01 to < 0.0001). parent BMPE and BMPE–Cur normalized ALT, AST, and ALP, with BMPE–Cur additionally restoring direct bilirubin but only partially correcting total bilirubin (*P* = 0.0183). The combination therapy almost normalized all hepatic markers and reduced ALP below sham levels (*P* < 0.0001), whereas the levan monotherapy normalized ALT and ALP, yielding lower ALT than BMPE (*P* = 0.0145) and lower ALP than DOX (*P* = 0.0009). However, serum levels of AST and direct BiL remained elevated compared with sham (*P* = 0.0016 and 0.003), BMPE–Cur and combination treatments (*P* < 0.01), as did the total BiL versus sham and the combination treatments (*P* < 0.0001 and 0.0002). In contrast, DOX failed to restore normal liver functions and showed inferior hepatoprotection relative to BMPE–Cur and the combination regimen despite the comparable outcomes to parent BMPE and levan monotherapies.

### Levan co-treatment enhances hepatic retention of BMPE–Cur nanocomplex

Quantitative LC–MS analysis demonstrated robust analytical performance, with linearity across 1–100 ppb and recovery values ranging from 95 to 109%. Curcumin was not detected in hepatic tissue in either nanocomplex-treated group. In contrast, the benzimidazole moiety (BMPE) was readily quantifiable following BMPE–Cur administration, whereas no corresponding signal was detected in mice receiving the parent BMPE formulation. Hepatic BMPE concentration increased from 2.352 ng/g in the BMPE–Cur group to 5.371 ng/g following co-administration with levan, representing a 2.3-fold increase in hepatic accumulation (*P* < 0.0001). To provide additional context for the observed biodistribution profile, in silico metabolism and transporter predictions were examined (Table 2). BMPE and curcumin were predicted to inhibit CYP2C19, CYP2C9, and CYP3A4, whereas fructose was predicted to inhibit CYP2C9 selectively. Curcumin was further predicted to act as a substrate for UDP-glucuronosyltransferase (UGT), sulfotransferase (SULT), and P-glycoprotein, while BMPE lacked predicted Phase II liabilities and was classified as a non-substrate for P-glycoprotein. Collectively, these findings demonstrate enhanced hepatic accumulation of BMPE following levan co-administration and provide a metabolic framework for subsequent mechanistic analyses.

**Table 2:** Predicted metabolic fate of key pharmacophores investigated in this study.

		BMPE	Curcumin	Fructose
Phase I Metabolism	CYP_2C19_inhibition	Inhibitor	Inhibitor	Non
	CYP_2C9_inhibition	Inhibitor	Inhibitor	Inhibitor
	CYP_2D6_inhibition	Non	Non	Non
	CYP_2D6_substrate	Non	Non	Non
	CYP_3A4_inhibition	Inhibitor	Inhibitor	Non
	CYP_3A4_substrate	Weakly	Non	Weakly
Phase II Metabolism	UGT	Non_substrate	Substrate	Non_substrate
	SULT	Non_substrate	Substrate	Non_substrate
Transporter	Pgp substrate classification	Non_substrate	Substrate	Non_substrate

***Abbreviations***: **CYP**: cytochrome P450; **UGT**: UDP-glucuronosyltransferase enzyme; **SULT**: sulfotransferase; **Pgp**: P-glycoprotein.

### Levan co-treatment enhances the antitumor and anti-angiogenic activity of BMPE–Cur

**Fig. 3:**
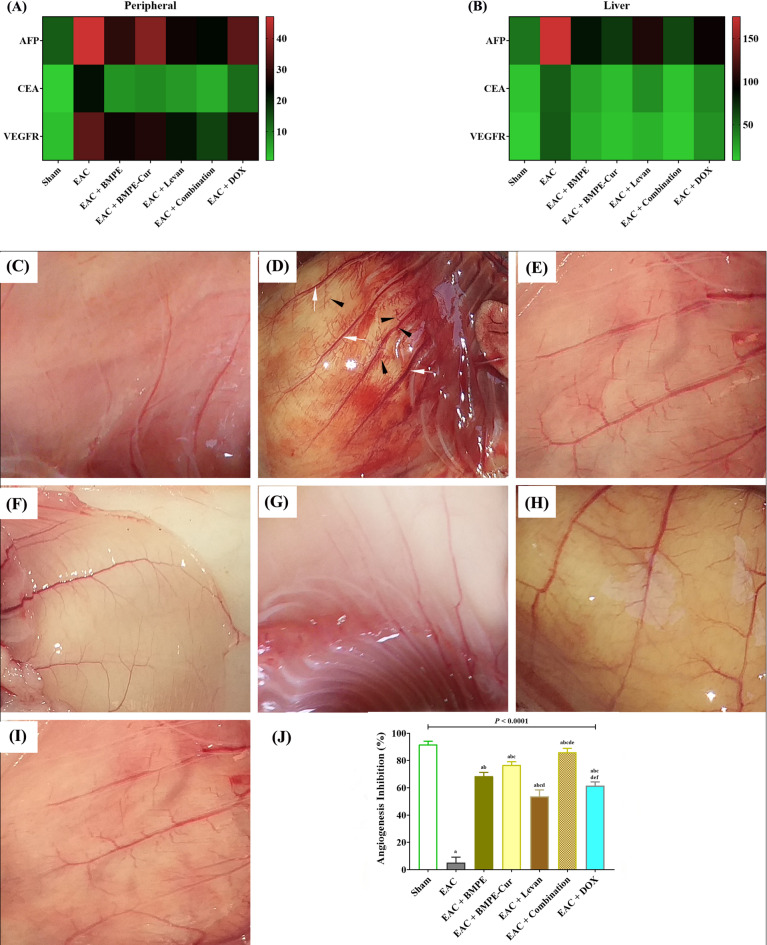
Effects of levan co-treatment on inhibition of peritoneal angiogenesis and tumor-associated biomarkers. Untreated mice showed marked elevations in circulating and hepatic AFP, CEA, and VEGFR versus sham (*P* < 0.0001, **A**,** B**). All treatments reduced these biomarkers, with the combination restoring hepatic AFP and VEGFR and circulating/hepatic CEA to near-baseline levels. BMPE–Cur ranked second and outperformed parent BMPE. The qualitative assessment revealed normal peritoneal vasculature in sham mice with few thin and orderly blood vessels visible, indicating the absence of pathological angiogenesis **(C)**. In contrast, the control EAC mice exhibited extensive pathological angiogenesis **(D)**, as indicated by numerous newly formed tortuous neovascularization radiating over the peritoneal surface **(black arrows)** and dilated blood vessels **(white arrows)**. Treatment with parent BMPE **(E)** resulted in a noticeable reduction in neovascularization compared to untreated EAC, where neovascularization appears less dense and less irregular, suggesting moderate inhibition of angiogenesis. Further inhibition of angiogenesis was observed in BMPE-Cur-treated mice **(F)** compared to the parent compound (*P* = 0.0061), as indicated by less dense, thinner, and less branched blood vessels, indicating a stronger anti-angiogenic effect. The most prominent anti-angiogenic effect was observed in EAC mice receiving the BMPE–Cur + levan combination **(G)**, with only a few fine, normal-like vessels observed. DOX treatment **(H)** significantly reduced neovascularization, with fewer and thinner vessels compared to untreated EAC, showing a less effective inhibition than parent BMPE (*P* = 0.009), BMPE–Cur alone, and in combination (*P* < 0.0001 for both). The levan monotherapy **(I)** presented the least effective anti-angiogenic effect compared with other treatments (*P* = 0.0033 – < 0.0001), showing a mild reduction in angiogenesis compared to untreated mice (–53.8 ± 4.66%), but vessels are still visible. Quantitative analysis **(J)** revealed significant inhibition of angiogenesis in all treated groups (*P* < 0.0001) compared with untreated EAC mice, with the combination treatment achieving the strongest anti-angiogenic effects. Data are mean ± SD. Statistical annotations: “a” vs. sham; “b” vs. EAC control; “c” vs. parent BMPE; “d” vs. BMPE–Cur; “e” vs. levan; “f” vs. combination.

The progression of EAC-induced hepatic tumorigenesis was associated with marked elevations in circulating and hepatic tumor-associated and angiogenic biomarkers compared with sham controls (Fig. 3A, B). Serum AFP, CEA, and VEGFR increased by 70.92 ± 2.22%, 97.17 ± 3.54%, and 93.18 ± 1.16%, respectively, while hepatic levels increased by 74.49 ± 1.41%, 77.32 ± 2.4%, and 84.26 ± 2.36% (*P* < 0.0001 for all). All therapeutic interventions significantly reduced these biomarkers relative to untreated EAC mice. Among the tested treatments, BMPE–Cur combined with levan produced the most pronounced effect, restoring AFP, CEA, and VEGFR to near-baseline levels and significantly outperforming free BMPE, DOX, and levan monotherapy. BMPE–Cur alone ranked second and consistently achieved greater suppression of tumor-associated biomarkers than free BMPE. Although DOX, free BMPE, and levan monotherapies also reduced biomarker levels, their effects were less pronounced and failed to normalize most parameters. Consistent with these findings, untreated EAC-bearing mice exhibited extensive peritoneal angiogenesis characterized by dense, tortuous, and highly branched neovasculature (Fig. 3D), whereas sham-operated animals displayed sparse and orderly vascular architecture (Fig. 3C). All treatments significantly inhibited angiogenesis compared with untreated EAC mice (*P* < 0.0001; Fig. 3J). Treatment with parent BMPE resulted in moderate inhibition of angiogenesis (–68.6 ± 2.7%), with a visible reduction in vessel density compared to the untreated group (Fig. 3E). Notably, the BMPE–Cur nanocomplex induced a more pronounced anti-angiogenic effect (–76.8 ± 2.39%), characterized by fewer and thinner blood vessels (Fig. 3F) than that in BMPE group (*P* = 0.0061). The combination of BMPE–Cur with levan produced the strongest suppression of angiogenesis (–86 ± 2.91%), outperforming both BMPE (*P* < 0.0001) and BMPE–Cur alone (*P* = 0.0005), where only sparse, fine vessels resembling normal vasculature were detected (Fig. 3G). Treatment with DOX (Fig. 3H) produced a significant reduction of neovascularization (–61.6 ± 2.7%), with fewer and thinner blood vessels compared to untreated EAC; however, this effect was weaker than that of parent BMPE (*P* = 0.009), BMPE–Cur alone, and in combination (*P* < 0.0001 for both). Levan monotherapy produced the least effective anti-angiogenic effect among all treatments (–53.8 ± 4.66%, *P* = 0.0033 – < 0.0001), showing visible but reduced vessel branching relative to untreated control (Fig. 3I).

### Levan co-treatment restores hepatic redox homeostasis

**Fig. 4:**
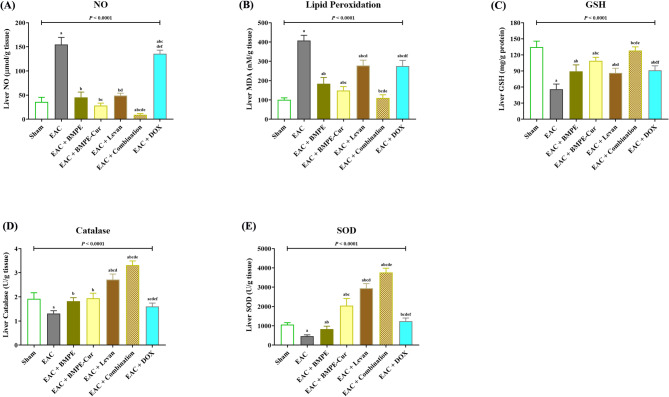
Effects of levan co-treatment on hepatic redox balance. Untreated EAC mice showed severe oxidative stress, with increased NO **(A)** and MDA **(B)** and depleted GSH **(C)**, catalase **(D)**, and SOD **(E)** (*P* < 0.0001). All interventions improved redox balance, with BMPE–Cur + levan producing the strongest antioxidant effect, normalizing MDA and GSH, reducing NO below sham levels, and elevating catalase and SOD above baseline (*P* < 0.0001). Data are mean ± SD. Statistical annotations: “a” vs. sham; “b” vs. EAC control; “c” vs. parent BMPE; “d” vs. BMPE–Cur; “e” vs. levan; “f” vs. combination.

Biochemical analysis of liver tissue revealed marked alterations in hepatic redox balance across the experimental groups. Relative to sham control, the untreated EAC animals exhibited significant elevation of liver nitrite (NO; +76.45 ± 3.29%; Fig. 4A) and lipid peroxidation (MDA; +74.57 ± 4.23%; Fig. 4B), accompanied by substantial depletion in endogenous. In parallel to metabolic dysfunction, biochemical analysis of liver tissue revealed marked alterations in hepatic redox balance across the experimental groups. Relative to sham control, the untreated EAC animals exhibited significant elevation of liver nitrite (NO; +76.45 ± 3.29%; Fig. 4A) and lipid peroxidation (MDA; +74.57 ± 4.23%; Fig. 4B), accompanied by substantial depletion in endogenous antioxidant defenses, including reduced glutathione (GSH; − 57.93 ± 3.88%; Fig. 4C), catalase (–34.15 ± 2.73%; Fig. 4D) and superoxide dismutase (SOD; − 58.34 ± 3.13%; Fig. 4E) (*P* < 0.0001/all markers). Except for DOX, which showed comparable catalase activity to the untreated animals, all other treatments significantly improved redox homeostasis, with the combination showing the strongest antioxidant effect. This regimen not only normalized oxidative damage markers (MDA: − 72.11 ± 4.64%), but elevated endogenous antioxidant defenses beyond physiological levels (GSH: +55.06 ± 2.73%; catalase: +60.31 ± 1.98%, and SOD: +87.74 ± 2.72% beyond sham levels, while suppressing NO below basal values (–94.04 ± 2.73%; *P* < 0.0001 vs. untreated mice; *P* = 0.0138 vs. sham). BMPE–Cur monotherapy exhibited comparable efficacy, normalizing NO (–81.54 ± 2.58%) and catalase (+ 27.21 ± 2.99%), and markedly enhancing SOD (+ 76.87 ± 4.45%, *P* < 0.0001). However, MDA remained elevated (*P* = 0.0055), and GSH was only partially restored (*P* = 0.0009), with no difference observed relative to the combination regarding catalase content. Other treatments significantly improved oxidative indices, reducing NO (–12.10% to − 70.65%) and MDA (–32.30% to − 57.47%) and increasing GSH (+ 34.96% to + 38.36%), catalase (+ 16.45% to + 51.55%), and SOD (+ 44.47% to + 84.41%) (*P* = 0.035 to < 0.0001), but none fully restored them to normal levels. The parent BMPE compound produced NO and GSH levels comparable to levan monotherapy but achieved more suppression of MDA levels (*P* = 0.0076), whereas the latter promoted catalase and SOD activities (*P* < 0.0001). Compared with DOX, all treatments except levan further reduced NO (*P* < 0.0001) and MDA (*P* = 0.0079 to < 0.0001), while BMPE–Cur and the combination significantly increased GSH (*P* = 0.017 and < 0.0001). Notably, the levan monotherapy uniquely enhanced catalase and SOD activities beyond DOX and the BMPE-Cur monotherapy (*P* < 0.0001).

### Levan co-treatment reprograms systemic and hepatic immune landscapes

**Fig. 5:**
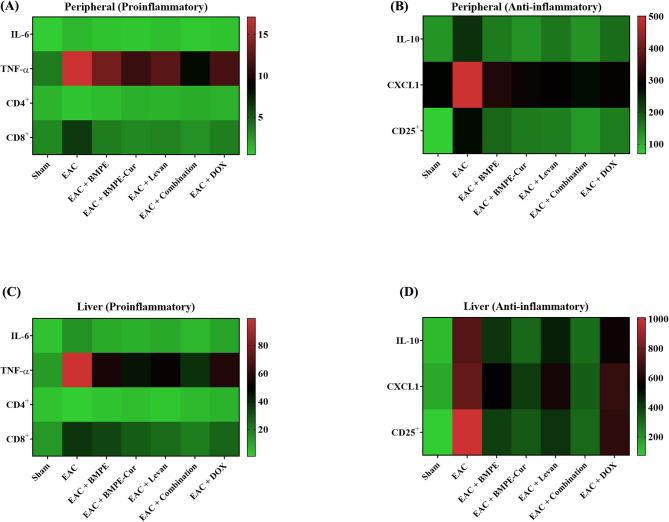
Heatmap representation showing the effects of different treatments on peripheral and hepatic immune markers in EAC mice. Heatmap analysis of peripheral and hepatic immune profiles in EAC mice. **(A**,** B)**: Untreated EAC mice exhibited peripheral immune dysregulation characterized by elevated IL-6, TNF-α, CD8⁺ T cells, IL-10, CXCL1, and CD25⁺ Tregs, with depletion of CD4⁺ T cells and reduced CD4⁺/CD8⁺ ratio (*P* < 0.0001). The combination therapy achieved a near-complete restoration of immune balance, normalizing cytokines, suppressing CD8⁺ and Tregs, and restoring the CD4⁺/CD8⁺ ratio. **(C**,** D)**: Hepatic immune profiling revealed severe inflammatory and immunosuppressive imbalance in untreated EAC mice. Combination therapy exerted the strongest correction, restoring helper T-cell dominance and suppressing inflammatory and regulatory mediators (*P* < 0.0001), outperforming all monotherapies. Data are mean ± SD.

Untreated EAC mice exhibited pronounced systemic immune dysregulation, reflected by significant elevations in circulating proinflammatory cytokines IL-6 and TNF-α (+ 62.97 ± 4.42% (*P* < 0.0001) and + 41.17 ± 4.87% (*P* = 0.0004), respectively) accompanied by marked depletion of CD4⁺ helper Th cells (–38.32 ± 7.06%) and expansion of CD8⁺ cytotoxic T cells (+ 48.71 ± 5.12%), resulting in a substantial reduction of the CD4⁺/CD8⁺ ratio by76.5 ± 2.59% (*P* < 0.0001; Fig. 5A). Concurrent increases in IL-10 (+ 43.40 ± 3.78%), CXCL1 (+ 41.34 ± 3.85%), and CD25⁺ regulatory T cells (+ 73.88 ± 3.75%) further indicated the establishment of a tumor-associated immunosuppressive milieu (*P* < 0.0001; Fig. 5B).

All therapeutic interventions partially mitigated these alterations; however, the magnitude of immune homeostasis differed markedly among treatment groups. BMPE–Cur monotherapy produced broad improvements, normalizing the cytokine levels and T-cell distributions, except for CD25⁺ Tregs (*P* < 0.0001 vs. sham), whereas co-administration with levan resulted in the most comprehensive correction of systemic immune dysregulation. This regimen normalized IL-6 (–48.91 ± 6.10%), TNF-α (–49.42 ± 5.19%), IL-10 (–42.53 ± 6.16%), CXCL1 (–43.54 ± 7.49%), CD8⁺ Tc cells (–54.55 ± 4.54%), and CD25⁺ Tregs (–57.90 ± 4.65%), while elevating CD4⁺ Th cells (+ 48.43 ± 4.46%) above sham levels, thereby restoring the CD4⁺/CD8⁺ ratio (all *P* < 0.0001). Monotherapies with free BMPE, levan, or DOX improved all immune indices but failed to fully re-establish immune equilibrium. Except for instances where BMPE–Cur monotherapy achieved comparable restoration of CD4⁺ T cells or IL-10 levels, comparative analyses confirmed that the combination regimen significantly outperformed all other treatments lowering systemic IL-6, TNF-α, CD8⁺ Tc cells, CXCL1, IL-10, and CD25⁺ Tregs (*P* = 0.0424–0.0014), and increasing CD4⁺ Th cells and the CD4⁺/CD8⁺ ratio (*P* = 0.0265 – < 0.0001). The levan monotherapy showed an immune profile broadly comparable to DOX, with slightly elevated IL-6 levels (*P* = 0.029), but the latter induced higher CD4⁺ counts than other treatments (*P* < 0.0001).

A similar pattern was observed at the hepatic level. Untreated EAC mice displayed severe hepatic immune dysregulation, characterized by marked elevation of IL-6 (+ 78.37 ± 3.46%), TNF-α (+ 87.30 ± 1.38%), CD8⁺ Tc cells (+ 63.17 ± 1.22%), IL-10 (+ 83.42 ± 3.13%), CXCL1 (+ 79.26 ± 3.11%), and CD25⁺ Tregs (+ 91.03 ± 3.67%), alongside profound depletion of CD4⁺ Th-cell depletion (–72.47 ± 3.89%), resulting in an 89.92 ± 1.07% reduction in the CD4⁺/CD8⁺ ratio (*P* < 0.0001 for all; Fig. 5C, D). While all treatments attenuated these disturbances, near-complete restoration of hepatic immune balance was achieved only with the combination regimen, showing the most pronounced suppression of IL-6 (–59.00 ± 6.55%), TNF-α (–61.10 ± 4.21%), IL-10 (–59.26 ± 3.44%), and CXCL1 (–67.30 ± 2.11%, *P* < 0.0001 for all). In parallel, this regimen presented the strongest recovery of CD4⁺ T cells (+ 82.63 ± 3.75%), while suppressing CD25⁺ Tregs (–69.63 ± 1.96%) and CD8⁺ Tc cells (–46.87 ± 1.76%), resulting in near-complete normalization of the hepatic CD4⁺/CD8⁺ ratio (+ 90.81 ± 1.69%; *P* < 0.0001 for all). Parent BMPE and levan monotherapies were less effective in correcting CD4⁺ depletion and regulatory T-cell expansion (*P* < 0.0001 and 0.003 vs. sham, respectively), but the nanocomplex alone exerted greater hepatic immunomodulatory effects than DOX; however, it remained inferior to the combination in restoring helper T-cell dominance (*P* < 0.0001). Relative to other treatments, the combination reduced inflammatory and immunosuppressive mediators by 15–50% (*P* = 0.0465 – < 0.0001) and increased CD4⁺ Th cells and CD4⁺/CD8⁺ ratios by 16–65% (*P* = 0.0065 – < 0.0001).

### Levan co-treatment reprograms inflammatory and metabolic signaling pathways

**Fig. 6:**
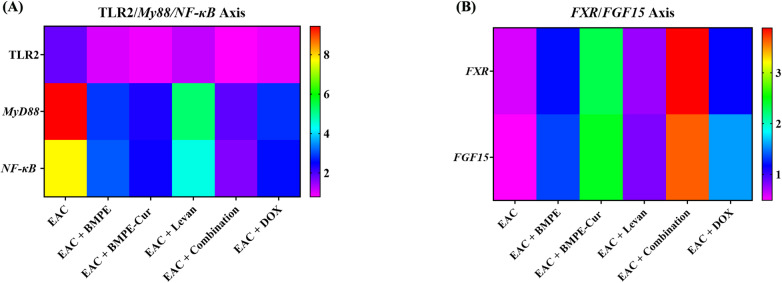
Heatmap representation of the effects of different treatments on hepatic TLR2-associated inflammatory signaling and *FXR*/*FGF15* metabolic regulation in EAC-bearing mice. Untreated EAC mice exhibited marked activation of TLR2/*MyD88*/*NF-κB* signaling **(A)**, evidenced by increased hepatic expression of TLR2 (1.81-fold), *MyD88* (9.43-fold), and *NF-κB* (7.74-fold), accompanied by significant suppression of *FXR* (− 41.1%) and *FGF15* (− 51.3%) expression **(B)** compared with sham controls (*P* < 0.0001 for all comparisons). All treatments partially reversed these alterations; however, BMPE–Cur combined with levan produced the most pronounced response, reducing TLR2, MyD88, and NF-κB expression by 56.47%, 80.05%, and 78.93%, respectively, while increasing FXR and FGF15 expression by 6.59- and 7.45-fold, respectively (*P* < 0.0001 vs. untreated EAC). BMPE–Cur monotherapy exhibited the second strongest effect, whereas DOX and parent BMPE produced intermediate responses. Levan monotherapy exerted the weakest overall modulation of both signaling axes, although partial restoration of pathway activity was observed. Comparative analyses demonstrated significantly greater suppression of TLR2-associated signaling and stronger restoration of *FXR*/*FGF15* expression in the combination group relative to all other treatments (*P* < 0.0001).

Untreated EAC mice exhibited marked activation of the TLR2-associated inflammatory pathway, evidenced by increased hepatic expression of TLR2 (+ 1.81-fold), *MyD88* (+ 9.43-fold), and *NF-κB* (+ 7.74-fold) relative to sham controls (Fig. 6A). This inflammatory phenotype was accompanied by significant suppression of the metabolic regulators *FXR* (–41.1%) and *FGF15* (–51.3%) (Fig. 6B; *P* < 0.0001 for all comparisons), indicating concurrent immune and metabolic dysregulation. All treatments partially reversed these alterations; however, the magnitude of improvement varied considerably among groups. The combination of BMPE–Cur and levan produced the most pronounced response, reducing TLR2, *MyD88*, and *NF-κB* expression by 56.47%, 80.05%, and 78.93%, respectively, while simultaneously increasing *FXR* and *FGF15* expression by 6.59- and 7.45-fold, respectively. These effects significantly exceeded those achieved by any monotherapy (*P* ≤ 0.0001). BMPE–Cur monotherapy ranked second, suppressing TLR2, *MyD88*, and *NF-κB* by 50.66%, 75.34%, and 68.45%, respectively, while increasing *FXR* and *FGF15* expression by 3.84-fold and 2.44-fold (*P* < 0.0001). DOX and parent BMPE produced intermediate effects, whereas levan monotherapy exhibited the weakest overall modulation of both signaling axes. Nevertheless, DOX, BMPE, and levan increased *FXR* expression by 1.96-fold, 2.02-fold, and 1.28-fold, respectively, and *FGF15* expression by 3.24-fold, 2.74-fold, and 1.70-fold, although the effect of levan on *FGF15* did not differ significantly from untreated EAC mice. Comparative analyses confirmed that the combination regimen achieved significantly greater inhibition of TLR2 (5.81–22.50%), *MyD88* (19.02–64.06%), and *NF-κB* (33.22–63.21%), together with significantly greater induction of *FXR* (40.13–80.52%) and *FGF15* (32.76–77.17%), than all other treatments (*P* < 0.0001). Notably, although BMPE–Cur produced TLR2 and *NF-κB* inhibition comparable to DOX, it surpassed the reference drug in suppressing *MyD88* expression and restoring *FXR* and *FGF15* expression (*P* < 0.0001 for all comparisons).

### Molecular docking supports direct interaction of BMPE and levan-derived ligands with TLR2

**Fig. 7:**
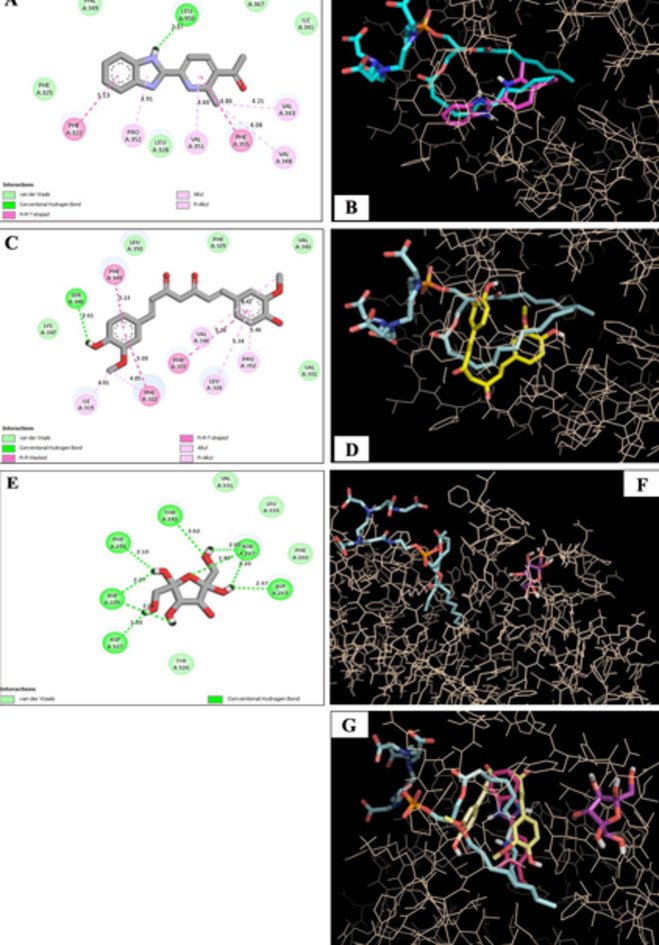
Predicted binding modes of key pharmacophores with TLR2. **(A)**: 2D-predicted binding mode of BMPE showing the types and distances of binding interactions within the TLR2 (PDB ID: 3A7C) binding pocket. **(B)**: Superimposition of BMPE **(pink)** with the native ligand PE-DTPA **(cyan)** in the TLR2 binding pocket, displayed as tinted sticks. **(C)**: 2D-predicted binding mode of curcumin (Cur) showing the types and distances of binding interactions within the TLR2 (PDB ID: 3A7C) binding pocket. **(D)**: Superimposition of Cur **(yellow)** with the native ligand PE-DTPA **(light blue)** in the TLR2 binding pocket, displayed as tinted sticks. **(E)**: 2D-predicted binding mode of fructose showing the types and distances of binding interactions within the TLR2 (PDB ID: 3A7C) binding pocket. **(F)**: 3D-predicted binding mode of fructose **(purple)** positioned adjacent to the native ligand PE-DTPA **(light blue)** in a newly identified side pocket of TLR2 (PDB ID: 3A7C), represented as tinted sticks. **(G)**: Combined 3D-predicted poses of BMPE **(pink)**, Cur **(yellow)**, and the native ligand PE-DTPA **(light blue)** within the main ligand-binding pocket, and fructose **(purple)** within a side pocket of TLR2 (PDB ID: 3A7C), all represented as tinted sticks.

Consistent with the observed suppression of hepatic TLR2 signaling, molecular docking analysis revealed favorable interactions between TLR2 and the tested ligands, including BMPE, curcumin, and fructose (Fig. 7). BMPE and curcumin exhibited the highest binding affinities, each yielding a docking score of − 7.0 kcal/mol, compared with − 6.5 kcal/mol for the native ligand PE-DTPA and − 5.9 kcal/mol for the reference agonist Pam2CSK4. Fructose displayed a lower binding affinity of − 5.5 kcal/mol. As shown in Fig. 7A and B, BMPE occupied the hydrophobic ligand-binding cavity of TLR2 and closely overlapped one of the lipid chains of the native ligand. The benzimidazole NH group formed a hydrogen bond with Leu350 (2.37 Å), while additional interactions included van der Waals and π–π contacts with Phe322 and Phe355, together with alkyl and π–alkyl interactions involving Val343, Val348, Val351, and Pro352. Curcumin exhibited a comparable binding orientation within the same cavity (Fig. 7C, D). A hydrogen bond was formed with Ser346 (2.61 Å), accompanied by van der Waals and π–π interactions with Phe322, Phe349, and Phe355, as well as alkyl and π–alkyl contacts involving Ile319, Leu328, Val348, and Pro352. Structural superimposition demonstrated overlap of curcumin with approximately one and a half of the two lipid chains of the native ligand. Fructose adopted a distinct binding orientation relative to BMPE and curcumin (Fig. 7E, F). The molecule formed nine hydrogen bonds with Asp263, Asn267, Asp294, Phe295, Asp327, and Thr330 and occupied an adjacent polar cavity. Among these residues, Asp327 was shared with the reported TLR2 ligand-binding site. The docking poses of BMPE, curcumin, and fructose within TLR2 are summarized in Fig. 7G.

**Fig. 8:**
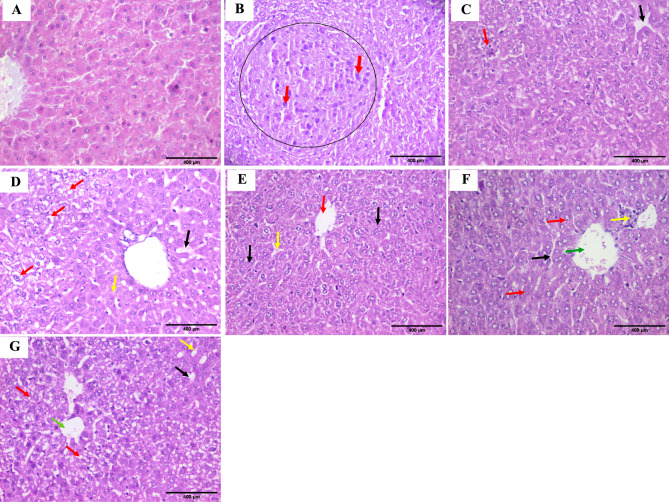
Histopathological examination of liver sections (H&E, scale bar = 400 μm) from experimental groups (*n* = 6–12). Sham control liver tissue **(A)** revealed preserved lobular architecture with well-arranged hepatocyte cords and intact sinusoids. Liver sections from untreated EAC control mice **(B)** showed marked architectural disruption, nuclear pleomorphism, increased cellular density, and necrotic foci **(red arrows within circled area)**. The free BMPE-treated group **(C)** presented partial restoration of hepatic cords with visible central vein **(red arrow)**; hepatocytes display reduced but persistent pleomorphism **(black arrows)**, with scattered degenerating cells **(yellow arrow)**. BMPE-Cur-treated group **(D)** showed marked histological improvement with preserved hepatocyte morphology, reduced necrotic changes **(red arrow)**, and well-defined vasculature **(black arrow)**. Combination (BMPE–Cur + levan) treatment **(E)** resulted in near-complete restoration of hepatic architecture, intact vascular structures **(black arrow)** and bile ducts **(yellow arrow)**, with minimal residual degeneration **(red arrows)**. DOX-treated group **(F)** showed improved lobular organization with a clear central vein **(green arrow)** and bile ducts **(yellow arrow)**; however, mild degenerative changes **(black/red arrows)** indicate residual hepatotoxicity. The levan-treated group **(G)** showed moderate histological improvement compared with the untreated control; hepatocytes show partial preservation but still exhibit degenerative changes **(red arrows)** and sinusoidal congestion **(black arrow)**.

### Histopathological confirmation of antitumor efficacy

As demonstrated in Fig. 8, histopathological examination of sham control liver tissue revealed preserved lobular architecture with well-arranged hepatocyte cords and intact sinusoids. In contrast, liver tissues revealed profound alterations in the untreated EAC control group, which exhibited complete disruption of hepatic architecture, dense cellular infiltration, pronounced nuclear pleomorphism, and necrotic foci, consistent with malignant progression. Treatment with the parent BMPE partially alleviated these alterations, restoring some hepatic cords and central veins, although residual degenerative changes and nuclear irregularities persisted. The BMPE–Cur nanocomplex produced a more pronounced improvement, characterized by preserved hepatocellular morphology, reduced necrosis, and well-defined sinusoidal spaces. Strikingly, the combination therapy demonstrated the most potent hepatoprotective effect, with near-normal lobular organization, intact vascular and biliary structures, and only minimal evidence of degeneration. Treatment with DOX significantly improved hepatic architecture; however, mild degenerative and congestive changes remained, reflecting its known hepatotoxic potential. In contrast, the levan monotherapy showed partial amelioration, with reduced but persistent degenerative changes and sinusoidal congestion, suggesting a supportive protective role rather than curative efficacy. Collectively, these findings indicate that the combination therapy exerts the strongest therapeutic effect, surpassing both single-drug regimens and probiotics alone. Comparative histopathological assessment of liver sections from control mice and different treated groups is summarized in Table 3.

**Table 3:** Comparative histopathological assessment of liver sections from untreated EAC-bearing mice and different treatment groups.

Group	Hepatic Architecture	Hepatocyte Morphology	Necrosis/Degeneration	Vascular & Sinusoidal Features	Overall Assessment
Sham control	Normal	Normal, well-arranged cords	Absent	intact vascular structures and sinusoids	preserved lobular architecture
Untreated EAC control	Severely disrupted; loss of lobular pattern	Marked nuclear pleomorphism, high cellular density	Extensive necrotic foci	Poorly defined, sinusoidal collapse	Severe malignant infiltration
Parent BMPE	Partially restored; cords visible	Reduced pleomorphism, but irregular nuclei persist	Focal degenerating hepatocytes	Central veins visible, mild congestion	Moderate protection
BMPE-Cur	Well-preserved lobular architecture	Hepatocytes polygonal with distinct nuclei, minimal atypia	Markedly reduced necrosis	Clear sinusoidal spaces, intact vascular structures	Strong hepatoprotection
Combination	Nearly normal organization	Regular hepatocytes with clear cytoplasm	Minimal degenerative changes	Intact central veins and bile ducts, well-defined sinusoids	Best therapeutic effect
DOX	Improved lobular pattern	Mostly normal hepatocytes, mild degenerative changes	Mild focal necrosis	Central veins and bile ducts preserved; slight congestion	Strong effect, but mild hepatotoxicity
Levan	Partially improved	Moderate preservation, some degenerative cells remain	Persistent degenerative/necrotic areas	Congested sinusoids, vascular changes	Supportive protective role

### Mathematical evaluation of BMPE–Cur and levan interaction profiles

Isobolographic analysis was performed to quantitatively assess the interaction between BMPE–Cur and levan across multiple biological endpoints (Supplementary Table S3). Combination index values indicated synergistic interactions (CI < 1) for the majority of evaluated parameters, particularly those associated with tumor burden, angiogenesis, oxidative stress, inflammatory signaling, and metabolic regulation. The strongest synergistic interactions were observed for angiogenic markers (VEGFR), tumor-associated biomarkers (AFP and CEA), inflammatory mediators including IL-6 and TNF-α, components of the TLR2/*MyD88*/*NF-κB* signaling pathway, and the *FXR*/*FGF15* metabolic axis. Synergistic antioxidant effects were also evident, as reflected by coordinated reductions in NO and MDA together with enhancement of endogenous antioxidant defenses. Immune-related endpoints displayed variable interaction profiles. Synergism was observed for CD8⁺ cytotoxic T cells, CD25⁺ regulatory T cells, and tissue inflammatory mediators. In contrast, partial antagonism was detected for selected systemic immune parameters, including serum IL-10 and peripheral CD4⁺ T cells, whereas hepatic IL-10 retained a synergistic interaction profile. Overall, the calculated combination indices demonstrated predominantly synergistic interactions between BMPE–Cur and levan across the evaluated tumor-associated, immunological, oxidative, and immunometabolic endpoints.

## Discussion

The development of targeted nanomedicines capable of overcoming rapid systemic clearance while simultaneously modulating the complex tumor microenvironment (TME) remains a major frontier in cancer therapeutics. The present study shows that microbial levan enhances the therapeutic efficacy of a PEGylated benzimidazole–curcumin (BMPE–Cur) nanocomplex in experimental liver cancer. Beyond improving antitumor activity, levan was associated with increased hepatic nanocomplex retention and coordinated immunometabolic remodeling, supporting the concept that modulation of host biological responses may complement conventional nanocarrier engineering to improve nanotherapeutic performance.

Consistent with the extensive tumor-associated hepatic injury characterized in this model^26^, the EAC-induced hepatic tumor model successfully recapitulated several hallmarks of advanced liver malignancy. These features included ascites accumulation, hepatic dysfunction, oxidative stress, inflammatory activation, angiogenesis, and profound immune dysregulation. The progressive increases in body weight and abdominal circumference, accompanied by elevated mortality, reflected a severe tumor burden^47^. Although all therapeutic interventions significantly improved survival and reduced EAC-associated morbidity, the combination of BMPE–Cur and levan consistently produced superior physiological outcomes. Interestingly, this combination achieved a greater reduction of abdominal circumference than the gold-standard chemotherapeutic doxorubicin (DOX) despite comparable body weights, suggesting more effective control of tumor-associated ascites and vascular permeability. Notably, the significant body-weight reduction observed in DOX-treated animals is consistent with previous reports describing DOX-induced systemic metabolic alterations associated with the depletion of adipose tissue and skeletal muscle mass^48^. In contrast, BMPE–Cur monotherapy did not induce significant body-weight loss despite its pronounced antitumor activity, while the co-administration of levan resulted in a moderate but significant reduction in body weight. This observation suggests that the microbial polysaccharide may have influenced the in vivo disposition of the nanocomplex, which in turn might have amplified BMPE-associated reduced food intake and body-weight loss upon its prolonged, sustained release^49^.

This pharmacodynamic hypothesis is supported by our pharmacokinetic findings, which revealed a 2.3-fold increase in hepatic BMPE accumulation with levan co-treatment. The limited residual hepatic concentrations of the parent BMPE compound likely reflect true metabolic clearance driven by tumor-induced oxidative stress and activation of conjugative enzymes, such as UDP-glucuronosyltransferase (UGTs) and sulfotransferase (SULTs), despite the predicted low physiological clearance of the compound^49^. In contrast, the absence of unmetabolized curcumin is consistent with its intrinsically rapid and extensive biotransformation through glucuronidation, sulfation, and reduction pathways, which is well documented even when nanoformulations improve its bioavailability^50^. Accordingly, while both compounds undergo metabolic clearance, the greater metabolic susceptibility of curcumin likely accounts for its complete absence at the terminal sampling point, whereas measurable hepatic BMPE concentrations remained detectable, particularly following levan co-administration. Importantly, the elevated hepatic BMPE levels resulting from the combination therapy suggests that levan may have altered the disposition of the nanocomplex and promoted the hepatic retention of the active payload. Our in silico metabolic predictions support this scenario, where fructose, the prominent monosaccharide unit of levan, was predicted to selectively inhibit cytochrome CYP2C9 while sparing CYP3A4 and CYP2C19, while concurrently attenuating P-glycoprotein efflux activity. Such selective modulation likely prolongs BMPE systemic exposure and enhances hepatic retention without inducing metabolic overload, while reduced drug efflux further consolidates local hepatic accumulation^7^.

The higher terminal hepatic BMPE concentration observed following levan co-administration was accompanied by substantial improvement in functional and structural indicators of tumor-associated liver injury. While BMPE–Cur alone significantly ameliorated the marked elevations in transaminases, bilirubin levels, cholestatic markers, tumor-associated biomarkers, and angiogenic mediators in EAC mice, co-administration with levan produced the most comprehensive restoration of hepatic function, nearly normalizing biochemical indices and markedly suppressing AFP, CEA, and VEGFR. These findings were further corroborated by the pronounced inhibition of peritoneal angiogenesis and the near-complete restoration of hepatic architecture observed histologically. While curcumin and benzimidazole scaffolds are known to inhibit VEGF/VEGFR signaling and promote endothelial apoptosis^51,52^, their combination with levan markedly amplified these effects, suggesting that the higher hepatic BMPE concentration observed with levan co-administration may have contributed to the superior antitumor efficacy observed at both biochemical and tissue levels. Interestingly, although DOX completely prevented mortality, it failed to restore hepatic function to the same extent as the combination treatment, consistent with its well-recognized hepatotoxic potential^53^.

Mechanistically, oxidative stress represents a major driver of hepatocarcinogenesis and tumor progression, contributing to genomic instability, chronic inflammation, angiogenesis, and hepatocellular injury^54^. The restoration of hepatic redox equilibrium by all treatments therefore likely contributed to the observed improvement in liver function and tumor-associated pathology^54,55^. Notably, the combination regimen produced the most profound antioxidant response, normalizing oxidative damage markers while elevating GSH, catalase, and SOD activities beyond baseline levels. These findings suggest activation of adaptive cytoprotective mechanisms, consistent with the potent antioxidant properties previously reported for BMPE^49^ and the well-established ability of curcumin to activate Nrf2-dependent antioxidant signaling^56^. The greater antioxidant response observed following levan co-administration is consistent with both the intrinsic antioxidant activity of levan observed in the present study and previous reports describing the antioxidant properties of microbial levans^14–16^. These findings suggest that levan contributed to the restoration of hepatic redox homeostasis. Given the close interplay between oxidative stress and inflammatory signaling^57^, the attenuation of oxidative injury may have also contributed to the downstream suppression of tumor-promoting pathways observed herein.

Given the established role of TLR2 signaling in promoting IL-10-producing Foxp3⁺CD25⁺ regulatory T-cell differentiation^58^, the observed reduction in Treg abundance is consistent with attenuation of TLR2-associated immunosuppressive signaling. At the signaling level, these immunological improvements were accompanied by pronounced suppression of the TLR2/*MyD88*/*NF-κB* signaling cascade, a key pathway linking gut-derived microbial signals to hepatic inflammation and tumor-promoting immune responses^59^. Interestingly, levan monotherapy exerted only modest suppression of hepatic TLR2/*MyD88*/*NF-κB* signaling, whereas its combination with BMPE–Cur produced the most pronounced inhibition of this pathway. This observation suggests that the principal contribution of levan is unlikely to involve direct inhibition of TLR2 signaling as the primary mechanism, despite previous reports suggesting inhibitory effects of β-(2→6)-type fructans on TLR2 signaling^60^. Instead, levan may exert an indirect modulatory effect through modulation of gut microbial communities that influence xenobiotic metabolism^61^, thereby influencing the pharmacokinetic disposition of the main pharmacophore (BMPE), as reflected by the higher terminal hepatic BMPE concentration observed following combination therapy. Although gut-derived microbial signals may contribute to this process, direct investigation of gut microbiota composition and function was beyond the scope of the present study.

Importantly, EAC-induced tumorigenesis was associated not only with activation of this inflammatory axis but also with concomitant suppression of *FXR* and *FGF15* expression, indicating parallel disruption of metabolic regulatory pathways^21^. While the combination treatment produced the strongest inhibition of TLR2-associated signaling, it simultaneously restored *FXR* and *FGF15* expression, suggesting coordinated immunometabolic remodeling^62^. Such dual regulation is particularly attractive from a therapeutic perspective because inflammatory and metabolic pathways are increasingly recognized as interconnected determinants of tumor progression, therapeutic responsiveness, and hepatic function^62,63^. Beyond restoring metabolic homeostasis, restoration of the *FXR*/*FGF15* pathway may also influence drug-metabolizing enzymes and transporter systems involved in xenobiotic disposition^22,23^, potentially contributing to the altered pharmacokinetic behavior reflected by the higher terminal hepatic BMPE concentration observed following combination therapy. These findings support the concept that optimizing the host biological environment may complement nanocarrier engineering as a strategy to improve nanotherapeutic performance.

To further explore the observed suppression of TLR2 signaling, molecular docking studies were performed to evaluate whether components of the therapeutic system could directly interact with the receptor. Both BMPE and curcumin displayed favorable binding affinities exceeding those of the reference TLR2 agonist Pam2CSK4 and comparable to the native ligand. Importantly, both molecules occupied the hydrophobic ligand-binding cavity and exhibited substantial overlap with the lipid chains of the co-crystallized ligand, suggesting the potential to interfere with receptor–ligand recognition^40,45^. These observations provide a plausible structural rationale consistent with the attenuation of TLR2-associated signaling observed in vivo. Interestingly, fructose, used as a representative structural unit of levan, also demonstrated measurable affinity toward TLR2 despite adopting a distinct binding orientation. Unlike BMPE and curcumin, fructose occupied an adjacent polar cavity stabilized predominantly through hydrogen-bond interactions. Although the intact three-dimensional structure of levan differs substantially from its constituent monosaccharides, these findings suggest that levan-derived structural motifs may contribute to TLR2-associated modulation. However, experimental validation would be required to determine whether these predicted interactions occur under physiological conditions and contribute to the biological effects observed in vivo.

**Fig. 9:**
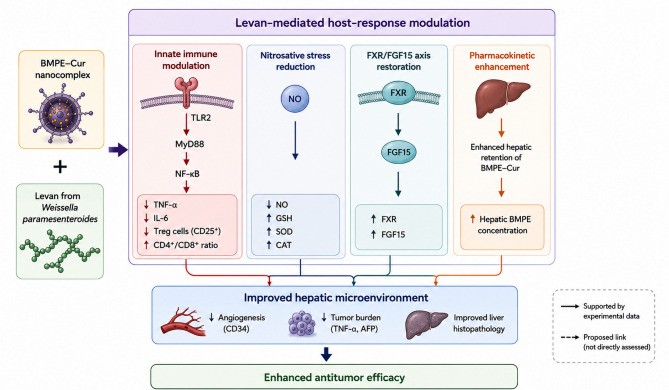
Proposed mechanistic framework of microbial levan-mediated potentiation of BMPE–Cur nanotherapeutic efficacy in experimental liver cancer. Co-administration of *W. paramesenteroides*-derived levan with the PEGylated benzimidazole–curcumin nanocomplex (BMPE–Cur) was associated with enhanced terminal hepatic BMPE retention, suppression of the TLR2/*MyD88*/*NF-κB* inflammatory pathway, and restoration of *FXR*/*FGF15* signaling. These coordinated immunometabolic changes were accompanied by attenuation of inflammatory and oxidative injury, inhibition of angiogenesis, restoration of immune homeostasis and hepatic function, and improved antitumor efficacy.

Histopathological observations corroborated the biochemical, immunological, and molecular findings, collectively supporting the superior therapeutic efficacy of the BMPE–Cur–levan combination. This integrated biological response was further substantiated by isobolographic analysis, which provided quantitative evidence that the enhanced efficacy of the combination treatment reflects genuine pharmacological synergy rather than a simple additive effect. Synergistic interactions predominated across tumor-associated biomarkers, angiogenesis, oxidative stress parameters, inflammatory mediators, components of the TLR2/*MyD88*/*NF-κB* pathway, and the *FXR*/*FGF15* axis, whereas antagonistic interactions were infrequent. This pattern suggests selective modulation of immune and metabolic pathways rather than broad, nonspecific enhancement. The broad distribution of synergistic effects across multiple pathological domains suggests that the therapeutic advantage of levan cannot be explained by modulation of a single biological pathway. Instead, levan appears to function as a host-response modulator capable of amplifying nanotherapeutic activity through coordinated regulation of interconnected oxidative, immune, inflammatory, and metabolic pathways. Collectively, these findings support a multifaceted mode of action that is consistent with the enhanced hepatic retention of BMPE and the comprehensive biological improvements observed throughout the study. The integrated mechanistic framework proposed based on the present findings is summarized schematically in Fig. 9.

### Limitations & future directions

Despite the strength and internal consistency of the present findings, several limitations should be acknowledged. First, although the study was designed to evaluate the biological consequences of levan co-administration, alterations in gut microbiota composition and microbial metabolite production were not directly assessed. Consequently, the contribution of microbiome remodeling to the observed immunometabolic effects remains inferential and warrants future investigation. Second, the pharmacokinetic evaluation was limited to hepatic tissue concentrations measured at a defined experimental endpoint. While these data clearly demonstrate enhanced hepatic accumulation of BMPE following levan co-administration, comprehensive pharmacokinetic characterization, including plasma exposure, systemic distribution, and temporal disposition profiles, was beyond the scope of the present study and should be addressed in future investigations. Third, although molecular docking and in silico metabolism analyses provided supportive mechanistic insight into potential TLR2 interactions and drug-metabolism pathways, these approaches remain predictive. Likewise, although restoration of *FXR*/*FGF15* signaling was strongly associated with enhanced therapeutic efficacy and increased hepatic BMPE accumulation, a direct causal relationship between this pathway and nanocomplex disposition was not experimentally established. Fourth, although the EAC-induced hepatic tumor model reproduces key features of tumor-associated hepatic pathology, it does not fully capture the molecular heterogeneity of human hepatocellular carcinoma.

Future studies incorporating receptor-binding assays, transporter activity measurements, and direct characterization of drug-metabolizing enzyme expression and activity, as well as targeted manipulation of *FXR*/*FGF15* signaling, will be required to confirm these mechanistic links. While the immune profiling captured the dominant immunological alterations in the EAC model, deeper characterization of innate immune populations, immune-cell polarization states, and functional T-cell responses would provide additional mechanistic resolution. Finally, future studies should define the therapeutic window of microbial levan through formal dose-escalation, mechanistic investigations into the reduced tolerability observed at higher doses, and long-term safety assessments. In addition, validation in complementary liver cancer models will be necessary before translational conclusions can be drawn.

## Conclusion

Taken together, these findings support the concept that microbial levan functions as more than a conventional prebiotic adjunct. Rather, it appears to function as a host-response modulator associated with enhanced nanotherapeutic retention and coordinated immunometabolic remodeling, characterized by suppression of TLR2/*MyD88*/*NF-κB* signaling and restoration of *FXR*/*FGF15* activity. The convergence of these pharmacokinetic and host-response effects provides a plausible mechanistic framework for the superior antitumor efficacy observed with the BMPE–Cur–levan combination. More broadly, the present work supports a paradigm in which microbial biopolymers may serve not only as adjunct therapeutic agents, but also as host-response modulators capable of reshaping the biological environment to favor nanotherapeutic retention and therapeutic response.

## Supplementary Information

Below is the link to the electronic supplementary material.

Supplementary Material 1

Supplementary Material 2

Supplementary Material 3

Supplementary Material 4

Supplementary Material 5

Supplementary Material 6

## Data Availability

Data supporting the findings of this study are available from the corresponding author upon reasonable request. No new reagents were generated in this study.

## Abbreviations

AFP: alpha-fetoprotein
ALB: albumin
ALT: alanine aminotransferase
AP: alkaline phosphatase
AST: aspartate aminotransferase
BiL: bilirubin
BMPE: 1-(6-(1 H-benzo[d]imidazol-2-yl)-2-methylpyridin-3-yl)ethanone
BSA: bovine serum albumin
CD: cluster of differentiation
CEA: carcinoembryonic antigen
CI: combination index
CXCL: C-X-C motif chemokine ligand
DOX: doxorubicin
EAC: Ehrlich ascites carcinoma
EPS: exopolysaccharide
FGF15: fibroblast growth factor 15
FXR: farnesoid X receptor
GAPDH: glyceraldehyde-3-phosphate dehydrogenase
GSH: glutathione reduced
HCC: Hepatocellular carcinoma
HIF-1α: hypoxia-inducible factor 1α
IL: interleukin
LAB: lactic acid bacteria
LC–MS: liquid chromatography- Mass Spectrometry
MAPK: mitogen-activated protein kinase
MDA: malondialdehyde
MDSCs: myeloid-derived suppressor cells
MyD88: myeloid differentiation factor 88
NF-κB: nuclear factor kappa B
NO: nitric oxide
PBS: phosphate-buffered saline
PE-DTPA: phosphatidylethanolamine-N-diethylenetriaminepentaacetic acid
Pgp: P-glycoprotein
PIGF: placental growth factor
RT: room temperature
RT-PCR: real-time polymerase chain reaction
SOD: superoxide dismutase
STAT3: Signal Transducer and Activator of Transcription 3
Tc: cytotoxic T cells
TGF-β: transforming growth factor-β
Th: T helper cells
TLR: toll-like receptor
TMB: 3,3′,5,5′-tetramethylbenzidine
TNF-α: tumor necrosis factor-α
Tregs: regulatory T cells
VEGFR: vascular endothelial growth factor

## References

[1] Chidambaranathan-Reghupaty, S., Fisher, P. B. & Sarkar, D. Hepatocellular carcinoma (HCC): Epidemiology, etiology and molecular classification. *Adv. Cancer Res.* **149**, 1–61. 10.1016/bs.acr.2020.10.001 (2021). 33579421 10.1016/bs.acr.2020.10.001 PMC8796122

[2] Anwanwan, D., Singh, S. K., Singh, S., Saikam, V. & Singh, R. Challenges in liver cancer and possible treatment approaches. *Biochim. Biophys. Acta Rev. Cancer* **1873**, 188314. 10.1016/j.bbcan.2019.188314 (2020). 31682895 10.1016/j.bbcan.2019.188314 PMC6981221

[3] Harini, A. & Perumal, I. Polymeric nanomaterials as a drug delivery system for anticancer and antibacterial infections: A review. *RSC Adv.* **15**, 32572–32592. 10.1039/d5ra01788b (2025). 40933086 10.1039/d5ra01788b PMC12417996

[4] Kumar, R. et al. Nanoparticle-based therapeutics for breast cancer: Advances, challenges, and clinical perspectives. *Curr. Pharm. Des.*10.2174/0113816128375291260226100138 (2026). 41937525 10.2174/0113816128375291260226100138

[5] Shawky, H., Fayed, D. B. & Ibrahim, N. E. pH-tailored delivery of a multitarget anticancer benzimidazole derivative using a PEGylated β-cyclodextrin-curcumin functionalized nanocomplex. *Biomater. Adv.* **163**, 213964. 10.1016/j.bioadv.2024.213964 (2024). 39053387 10.1016/j.bioadv.2024.213964

[6] Ibrahim, N. E., Shawky, H., Maghraby, A. S. & Farrag, E. K. Insights into the pharmacokinetics, biodistribution, and oral toxicity of a polymeric benzimidazole - curcumin nanocomplex with a multitarget anticancer potential. *Food Chem. Toxicol.* **202**, 115483. 10.1016/j.fct.2025.115483 (2025). 40312001 10.1016/j.fct.2025.115483

[7] Luo, F. et al. Polymeric nanomedicines for the treatment of hepatic diseases. *J. Nanobiotechnol.* **20**, 488. 10.1186/s12951-022-01708-y (2022). 10.1186/s12951-022-01708-y PMC9675156 36402994

[8] Perrigue, P. M., Murray, R. A., Mielcarek, A., Henschke, A. & Moya, S. E. Degradation of drug delivery nanocarriers and payload release: A review of physical methods for tracing nanocarrier biological fate. *Pharmaceutics* **13**, 770. 10.3390/pharmaceutics13060770 (2021). 34064155 10.3390/pharmaceutics13060770 PMC8224277

[9] Ma, Z. F., Fu, C. & Lee, Y. Y. The modulatory role of bioactive compounds in functional foods on inflammation and metabolic pathways in chronic diseases. *Foods* **14**, 821. 10.3390/foods14050821 (2025). 40077524 10.3390/foods14050821 PMC11899172

[10] Waoo, A. A. et al. Microbial exopolysaccharides in the biomedical and pharmaceutical industries. *Heliyon* **9**, e18613. 10.1016/j.heliyon.2023.e18613 (2023). 37593641 10.1016/j.heliyon.2023.e18613 PMC10432183

[11] Kiran, N. S., Yashaswini, C., Singh, S. & Prajapati, B. G. Revisiting microbial exopolysaccharides: A biocompatible and sustainable polymeric material for multifaceted biomedical applications. *3 Biotech* **14**, 95. 10.1007/s13205-024-03946-3 (2024). 38449708 10.1007/s13205-024-03946-3 PMC10912413

[12] Young, I. D., Latousakis, D. & Juge, N. The Immunomodulatory Properties of β-2,6 Fructans. *Compr. Rev. Nutrients*. **13**, 1309. 10.3390/nu13041309 (2021). 10.3390/nu13041309 PMC8071392 33921025

[13] Peled, E. et al. First transcriptomic insight into the reprogramming of human macrophages by levan-type fructans. *Carbohydr. Polym.* **320**, 121203. 10.1016/j.carbpol.2023.121203 (2023). 37659791 10.1016/j.carbpol.2023.121203

[14] Saleh, S. A. A. et al. Prebiotic-mediated gastroprotective potentials of three bacterial levans through NF-κB-modulation and upregulation of systemic IL-17A. *Int. J. Biol. Macromol.* **250**, 126278. 10.1016/j.ijbiomac.2023.126278 (2023). 37572818 10.1016/j.ijbiomac.2023.126278

[15] Shawky, H. et al. Dose-dependent antiulcerogenic effect of bacterial levans on indomethacin-induced gastric lesions through modulation of NF-κB/TNF-α expression and apoptotic pathways. *Int. J. Biol. Macromol.* **330**, 148143. 10.1016/j.ijbiomac.2025.148143 (2025). 41067344 10.1016/j.ijbiomac.2025.148143

[16] Shawky, H. et al. Immunotherapeutic effects of *de novo* benzimidazole derivative and prebiotic bacterial levan against triple-negative breast tumors by harnessing the immune landscape to intercept the oncogenic transcriptome. *Int. J. Biol. Macromol.* **289**, 138844. 10.1016/j.ijbiomac.2024.138844 (2025). 39701264 10.1016/j.ijbiomac.2024.138844

[17] Hu, Y., Gao, M., Chenghuang, J. & Bao, R. Insights into the gut-liver axis: Mechanisms and emerging therapies in hepatocellular carcinoma. *Front. Pharmacol.* **16**, 1595853. 10.3389/fphar.2025.1595853 (2025). 40458800 10.3389/fphar.2025.1595853 PMC12127201

[18] Qian, C., Cao, X. & Hoffmann, J. A. Toll-like receptors in innate immunity and inflammation: from fundamental biology to clinic insights. *Immun. Inflamm.* **2**, 22. 10.1007/s44466-026-00040-6 (2026). 42052154 10.1007/s44466-026-00040-6 PMC13111532

[19] Di Lorenzo, A., Bolli, E., Tarone, L., Cavallo, F. & Conti, L. Toll-like receptor 2 at the crossroad between cancer cells, the immune system, and the microbiota. *Int. J. Mol. Sci.* **21**, 9418. 10.3390/ijms21249418 (2020). 33321934 10.3390/ijms21249418 PMC7763461

[20] Zhou, M. et al. From gut to liver: Exploring the crosstalk between gut-liver axis and oxidative stress in metabolic dysfunction-associated steatotic liver disease. *Ann. Hepatol.* **30**, 101777. 10.1016/j.aohep.2025.101777 (2025). 39832564 10.1016/j.aohep.2025.101777

[21] Katafuchi, T. & Makishima, M. Molecular basis of bile acid-FXR-FGF15/19 signaling axis. *Int. J. Mol. Sci.* **23**, 6046. 10.3390/ijms23116046 (2022). 35682726 10.3390/ijms23116046 PMC9181207

[22] Maliha, S. & Guo, G. L. Farnesoid X receptor and fibroblast growth factor 15/19 as pharmacological targets. *Liver Res.* **5**, 142–150. 10.1016/j.livres.2021.02.002 (2021). 39957843 10.1016/j.livres.2021.02.002 PMC11791795

[23] Rizzolo, D. et al. Effects of overexpression of fibroblast growth factor 15/19 on hepatic drug metabolizing enzymes. *Drug Metab. Dispos.* **50**, 468–477. 10.1124/dmd.121.000416 (2022). 34965924 10.1124/dmd.121.000416 PMC11022908

[24] Wen, Y. Q. et al. FXR activation remodels hepatic and intestinal transcriptional landscapes in metabolic dysfunction-associated steatohepatitis. *Acta Pharmacol. Sin.* **45**, 2313–2327. 10.1038/s41401-024-01329-1 (2024). 38992119 10.1038/s41401-024-01329-1 PMC11489735

[25] Xu, J., Sun, P., Zhu, W., Liu, X. & Ma, L. FGF19 in solid tumors: Molecular mechanisms, metabolic reprogramming, and emerging therapeutic opportunities. *Theranostics* **16**, 345–397. 10.7150/thno.121601 (2026). 41328353 10.7150/thno.121601 PMC12665127

[26] Dos Santos, D. C. et al. Apoptosis oxidative damage-mediated and antiproliferative effect of selenylated imidazo[1,2-a]pyridines on hepatocellular carcinoma HepG2 cells and in vivo. *J. Biochem. Mol. Toxicol.* **35**, e22663. 10.1002/jbt.22663 (2021). 33125183 10.1002/jbt.22663

[27] Abd El-Karim, S. S., Anwar, M. M., Zaki, E. R., Elseginy, S. A. & Nofal, Z. M. Synthesis and molecular modeling of new benzimidazoles as glutathione S-transferase inhibitors and anticancer agents. *Future Med. Chem.* **10**, 157–181. 10.4155/fmc-2017-0137 (2018). 29235906 10.4155/fmc-2017-0137

[28] Amer, M. N. et al. Structure elucidation and in vitro biological evaluation of sulfated exopolysaccharide from LAB *Weissella paramesenteroides* MN2C2. *J. Appl. Pharm. Sci.* **11**, 022–31. 10.7324/JAPS.2021.110504 (2021).

[29] Medhat, D. et al. Effect of Au-dextran NPs as anti-tumor agent against EAC and solid tumor in mice by biochemical evaluations and histopathological investigations. *Biomed. Pharmacother.* **91**, 1006–1016. 10.1016/j.biopha.2017.05.043 (2017). 28525943 10.1016/j.biopha.2017.05.043

[30] Meng, C. et al. Preparation and evaluation of animal models of cardiotoxicity in antineoplastic therapy. *Oxid. Med. Cell Longev.* **2022**, 3820591. 10.1155/2022/3820591 (2022). 35847594 10.1155/2022/3820591 PMC9277159

[31] Noaman, E., Badr El-Din, N. K., Bibars, M. A., Abou Mossallam, A. A. & Ghoneum, M. Antioxidant potential by arabinoxylan rice bran, MGN-3/biobran, represents a mechanism for its oncostatic effect against murine solid Ehrlich carcinoma. *Cancer Lett.* **268**, 348–59. 10.1016/j.canlet.2008.04.012 (2008). 18554778 10.1016/j.canlet.2008.04.012

[32] Hwang, S. et al. An in silico phase I and II drug metabolism prediction platform. *Drug Metab. Pharmacokinet.* **35**, 361–367. 10.1016/j.dmpk.2020.05.007 (2020). 32616370 10.1016/j.dmpk.2020.05.007

[33] Hajra, S., Patra, A. R., Basu, A., Saha, P. & Bhattacharya, S. Indole-3-carbinol (I3C) enhances the sensitivity of murine breast adenocarcinoma cells to doxorubicin (DOX) through inhibition of NF-κβ, blocking angiogenesis and regulation of mitochondrial apoptotic pathway. *Chem, Biol, Interact.* **290**, 19–36. 10.1016/j.cbi.2018.05.005 (2018). 29775573 10.1016/j.cbi.2018.05.005

[34] Engvall, E. & Perlmann, P. Enzyme-linked immunosorbent assay (ELISA). Quantitative assay of immunoglobulin G. *Immunochemistry* **8**, 871–874. 10.1016/0019-2791(71)90454-x (1971). 5135623 10.1016/0019-2791(71)90454-x

[35] Razin, M. et al. TLR3\TLR7 as differentially expressed markers among viral, nonviral, and autoimmune diseases in Egyptian patients. *Viral Immunol.* **34**, 607–621. 10.1089/vim.2021.0006 (2021). 34342515 10.1089/vim.2021.0006

[36] Feng, Y., Zou, L., Chen, C., Li, D. & Chao, W. Role of cardiac- and myeloid-MyD88 signaling in endotoxin shock: a study with tissue-specific deletion models. *Anesthesiology* **121**, 1258–1269. 10.1097/ALN.0000000000000398 (2014). 25089642 10.1097/ALN.0000000000000398 PMC4237623

[37] Li, G. et al. Farnesoid X receptor induces murine scavenger receptor class B type I via intron binding. *PLoS One* **7**, e35895. 10.1371/journal.pone.0035895 (2012). 22540009 10.1371/journal.pone.0035895 PMC3335076

[38] Thomas, A. M. et al. Genome-wide tissue-specific farnesoid X receptor binding in mouse liver and intestine. *Hepatology* **51**, 1410–1419. 10.1002/hep.23450 (2010). 20091679 10.1002/hep.23450 PMC4855519

[39] Suvarna, S. K. & Layton, C. The gross room/surgical cut-up. In *Bancroft’s Theory and Practice of Histological Techniques* 7th Edition (eds Suvarna, S. K. et al.) 95–103 (Churchill Livingstone, 2013).

[40] Kang, J. Y. et al. Recognition of lipopeptide patterns by Toll-like receptor 2-Toll-like receptor 6 heterodimer. *Immunity* **31**, 873–884. 10.1016/j.immuni.2009.09.018 (2009). 19931471 10.1016/j.immuni.2009.09.018

[41] Trott, O. & Olson, A. J. AutoDock Vina: improving the speed and accuracy of docking with a new scoring function, efficient optimization, and multithreading. *J. Comput. Chem.* **31**, 455–461. 10.1002/jcc.21334 (2010). 19499576 10.1002/jcc.21334 PMC3041641

[42] Ali, E. A. et al. Synthesis and computational investigation of novel 2-mercaptoimidazolones as dual antimicrobial and anti-proliferative agents with potential multitargeting kinase inhibitory activity. *Sci. Rep.* **15**31527. 10.1038/s41598-025-17260-2 (2025). 40866510 10.1038/s41598-025-17260-2 PMC12391495

[43] El-Hussieny, M. et al. Iron (III) triflate, a new efficient catalyst for the synthesis of new bis (oxy)-bis (phenylene)-ethane-Schiff bases: In vitro antioxidant, anti-inflammatory, α-amylase, α-glucosidase biochemical screening, and in silico study. *J. Mol. Struct.* **1349**, 143627. 10.1016/j.molstruc.2025.143627 (2026).

[44] Krieger, E. et al. Improving physical realism, stereochemistry, and side-chain accuracy in homology modeling: Four approaches that performed well in CASP8. *Proteins* **77**, 114–122. 10.1002/prot.22570 (2009). 19768677 10.1002/prot.22570 PMC2922016

[45] Koymans, K. J. et al. Structural basis for inhibition of TLR2 by staphylococcal superantigen-like protein 3 (SSL3). Proc. Natl. Acad. Sci. USA. 112, 11018–11023 (2015). 10.1073/pnas.1502026112 10.1073/pnas.1502026112 PMC4568226 26283364

[46] Chou, T. C. & Martin, N. CompuSyn for drug combinations. A Computer software for quantitation of synergism and antagonism, and the determination of IC50, ED50 and LD50 valuesComboSyn, Paramus, NJ, (2005). www.combosyn.com

[47] Gowda, N. G. S. et al. Ehrlich Ascites carcinoma mice model for studying liver inflammation and fibrosis. *Adv. Cancer Biol-Metastasis*. **4**, 100029. 10.1016/j.adcanc.2022.100029 (2022).

[48] Cella, P. S. et al. Doxorubicin causes cachexia, sarcopenia, and frailty characteristics in mice. *PLoS One* **19**, e0301379. 10.1371/journal.pone.0301379 (2024). 38648220 10.1371/journal.pone.0301379 PMC11034664

[49] Farrag, E. K., Aziz, W. M., Shaker, S. E., Shawky, H. & Fayed, D. B. Toxicological profiling of a de novo synthesized benzimidazole derivative with potent and selective proapoptotic potentials against breast cancer. *Food Chem. Toxicol.* **180**, 114049. 10.1016/j.fct.2023.114049 (2023). 37734466 10.1016/j.fct.2023.114049

[50] Li, N. et al. Comparative pharmacokinetic assessment of curcumin in rats following intratracheal instillation versus oral administration: Concurrent detection of curcumin and its conjugates in plasma by LC-MS/MS. *Pharmaceutics* **16**, 1459. 10.3390/pharmaceutics16111459 (2024). 39598582 10.3390/pharmaceutics16111459 PMC11597260

[51] Yassen, A. S. A. et al. Novel curcumin-based analogues as potential VEGFR2 inhibitors with promising metallic loading nanoparticles: synthesis, biological evaluation, and molecular modelling investigation. *RSC Med. Chem.* **15**, 4039–4067. 10.1039/d4md00574k (2024). 39345715 10.1039/d4md00574k PMC11428034

[52] Elkady, H. et al. New benzoxazole derivatives as potential VEGFR-2 inhibitors and apoptosis inducers: Design, synthesis, anti-proliferative evaluation, flow cytometric analysis, and in silico studies. *J. Enzyme Inhib. Med. Chem.* **37**, 397–410. 10.1080/14756366.2021.2015343 (2022). 34961427 10.1080/14756366.2021.2015343 PMC8725875

[53] Soltani Hekmat, A., Hekmat, M., Ramezanipour, S. & Javanmardi, K. Protective effects of Alamandine against doxorubicin-induced liver injury in rats. *BMC Pharmacol. Toxicol.* **26**, 95. 10.1186/s40360-025-00932-0 (2025). 40319302 10.1186/s40360-025-00932-0 PMC12048956

[54] Li, Y., Yu, Y., Yang, L. & Wang, R. Insights into the Role of oxidative stress in hepatocellular carcinoma development. *Front. Biosci.* **28**, 286. 10.31083/j.fbl2811286 (2023). 10.31083/j.fbl2811286 38062825

[55] Allameh, A., Niayesh-Mehr, R., Aliarab, A., Sebastiani, G. & Pantopoulos, K. Oxidative stress in liver pathophysiology and disease. *Antioxidants* **12**, 1653. 10.3390/antiox12091653 (2023). 37759956 10.3390/antiox12091653 PMC10525124

[56] Park, J. Y., Sohn, H. Y., Koh, Y. H. & Jo, C. Curcumin activates Nrf2 through PKCδ-mediated p62 phosphorylation at Ser351. *Sci. Rep.* **11**, 8430. 10.1038/s41598-021-87225-8 (2021). 33875681 10.1038/s41598-021-87225-8 PMC8055680

[57] Liu, Z. Y., Yu, Y. & Yu, X. Z. Interplay between oxidative stress and inflammation in aquatic animals: Mechanisms, consequences, and implications for aquaculture health. *Antioxidants.* **15**, 208. 10.3390/antiox15020208 (2026). 41750589 10.3390/antiox15020208 PMC12938112

[58] Cosovanu, C. & Neumann, C. The many functions of Foxp3^+^ regulatory T cells in the intestine. *Front. Immunol.* **11**, 600973. 10.3389/fimmu.2020.600973 (2020). 33193456 10.3389/fimmu.2020.600973 PMC7606913

[59] Chen, H. et al. Differential regulatory mechanisms of gut-derived SCFAs (acetate, propionate, butyrate) on the pulmonary TLR2 signaling pathway and their roles in the inflammatory balance of bacterial pneumonia. *Eur. J. Med. Res.* **31**, 249. 10.1186/s40001-025-03666-1 (2026). 41521304 10.1186/s40001-025-03666-1 PMC12882167

[60] Akkerman, R. et al. Exopolysaccharide β-(2,6)-levan-type fructans have a molecular-weight-dependent modulatory effect on Toll-like receptor signalling. *Food Funct.* **15**, 676–688. 10.1039/d3fo03066k (2024). 38108152 10.1039/d3fo03066k PMC10802977

[61] Mousa, S., Sarfraz, M. & Mousa, W. K. The interplay between gut microbiota and oral medications and its impact on advancing precision medicine. *Metabolites* **13**, 674. 10.3390/metabo13050674 (2023). 37233715 10.3390/metabo13050674 PMC10221081

[62] Marbaniang, C., Kma, L. & Sharan, R. N. Immunometabolism in the tumor microenvironment: Dual role in modulating anti-tumor immunity and tumor progression. *Asian Pac. J. Cancer Prev.* **26**, 3881–3893. 10.31557/APJCP.2025.26.11.3881 (2025). 41312912 10.31557/APJCP.2025.26.11.3881 PMC12927519

[63] Jee, Y. M., Lee, J. Y. & Ryu, T. Chronic inflammation and immune dysregulation in metabolic-dysfunction-associated steatotic liver disease progression: From steatosis to hepatocellular carcinoma. *Biomedicines* **13**, 1260. 10.3390/biomedicines13051260 (2025). 40427086 10.3390/biomedicines13051260 PMC12109540

