# Supplementary material for: Microbial levan potentiates hepatic retention and antitumor activity of a PEGylated benzimidazole–curcumin nanocomplex through TLR2–FXR/FGF15-associated immunometabolic remodeling in experimental liver cancer
Source: Sci Rep. 2026 Aug 12;16:25094. doi: 10.1038/s41598-026-64576-8 (PMC13462577; doi:10.1038/s41598-026-64576-8)
Supplement: Supplementary file 1 [file 41598_2026_64576_MOESM6_ESM.docx]

**Microbial Levan Potentiates Hepatic Retention and Antitumor Activity of a PEGylated Benzimidazole–Curcumin Nanocomplex through TLR2–*FXR*/*FGF15*-Associated Immunometabolic Remodeling in Experimental Liver Cancer**

**Heba Shawky****<https://orcid.org/0000-0001-8403-1143>^*^, Noha E. Ibrahim****<https://orcid.org/0000-0002-7176-4687>, Mai N. Amer****<https://orcid.org/0000-0002-9881-8382>, Aisha A.K. Al-Ashmawy****<https://orcid.org/0000-0002-9219-5436>, Olfat A. Hammam****^<https://orcid.org/0000-0002-4965-5804>^, Dalia B. Fayed****<https://orcid.org/0000-0001-5238-7516>, Amany S. Maghraby****<https://orcid.org/0000-0002-9723-5011>**

**Table of Contents**

**Supplementary Results**

Structural confirmation of microbial levan	2

*FT-IR analysis*	2

*NMR spectroscopic analysis*	2

*UV-Vis spectrum*	3

**Supplementary Figures**

Fig. S1: Determination of sample size using G-Power software	4

Fig. S2: FT-IR spectrum of the purified levan	5

Fig. S3: ^1H NMR spectrum of the purified levan recorded in *DMSO-d₆*	6

Fig. S4: UV–visible absorption spectrum of the purified levan	7

**Supplementary Tables**

Supplementary Table 1. Optimized parameters for the LC-ESI-MS/MS analysis of parent (unmetabolized) BMPE and curcumin concentrations in EAC liver homogenates	8

Supplementary Table 2. Primer sets used for profiling the inflammatory and metabolic gene expression in EAC livers	9

Supplementary Table 3. Isobolographic analysis of BMPE-Cur – levan interactions	10

**Supplementary References**	12

**Supplementary Results**

**Structural confirmation of microbial levan**

***FT-IR analysis***

The functional groups of the purified levan were examined by FT-IR spectroscopy **(Fig. S2)**. The spectrum exhibited a broad and intense absorption band at approximately 3438 cm⁻¹, characteristic of O–H stretching vibrations of hydroxyl groups typical of polysaccharides **[S1]**, which indicate extensive hydrogen bonding within the polysaccharide matrix. Weak bands observed at 2929 and 2841 cm⁻¹ correspond to asymmetric and symmetric C–H stretching vibrations of aliphatic –CH₂ groups commonly associated with sugar ring structures **[14]**. A band at 1637 cm⁻¹ was primarily attributed to H–O–H bending vibrations of bound water molecules, a common feature of hydrophilic exopolysaccharides **[S2]**, although minor contributions from carbonyl-containing residues cannot be entirely excluded. In the fingerprint region (1500–500 cm⁻¹), the absorption at 1444 cm⁻¹ is assigned to C–H bending and O–H deformation modes of carbohydrate residues **[S3]**. The prominent bands at 1142 cm⁻¹ and 976 cm⁻¹ correspond to C–O–C glycosidic linkages and furanose ring vibrations, consistent with fructan-type polysaccharides **[14, S1, S2]**. Weak absorptions were detected in the 1250–1300 cm⁻¹ region and near 555 cm⁻¹, which has been reported in some studies to overlap with sulfate ester S=O stretching vibrations and O–S–O bending vibrations, respectively **[S4]**. However, these signals may arise from trace sulfate-associated residues originating from the fermentation medium, given the low sulfur content previously determined (0.78%, corresponding to a low degree of sulfation (approximately 0.04–0.045 sulfate groups/monosaccharide unit) **(S2)**, and the absence of definitive covalent sulfation. Overall, the FT-IR spectral profile is consistent with a fructose-dominated polysaccharide characterized by β-linked glycosidic bonds **[14, S2, S3]**, supporting classification of the polymer as a levan-type fructan.

***NMR spectroscopic analysis***

The ^1H NMR spectrum of the bacterial polysaccharide displayed characteristic features of a carbohydrate polymer **(Fig. S3)**. The spectrum was dominated by a broad and intense envelope of signals between 3.6 and 4.4 ppm, corresponding to ring protons (H-3, H-4, H-5, and H-6) of fructofuranosyl residues **[14]**, consistent with the fructose-rich composition determined by monosaccharide analysis **[S2]**. Weak signals observed in the region of 4.9–5.4 ppm were attributed to anomeric protons of minor α-D-glucopyranosyl residues **[13, S5]**, likely representing terminal glucose units derived from sucrose, the substrate for levansucrase- mediated fructan biosynthesis in lactic acid bacteria **[14, 61, S6]**. The low intensity of these anomeric signals correlates with the previously reported minor glucose content (9%) **[S2]**, and supports the predominance of fructose-based repeating units within the polymer backbone **[14, 61]**. Broad resonances observed between 4.1 and 5.1 ppm are consistent with glycosidic linkages and substituted carbohydrate environments commonly reported for β-linked fructans **[14, S6]**. However, these downfield shifts are not uniquely diagnostic of specific chemical modifications and may reflect overlapping signals arising from branching architecture or hydrogen-bonding interactions within the polymer matrix **[S7]**. Minor signals detected between 0.9 and 2.4 ppm were of low intensity and may arise from trace acetyl groups from glucosamine **[S8]**, but their presence, particularly in relatively low intensity, does not indicate major structural modification of the polysaccharide backbone. Overall, the ^1H NMR spectral characteristics, in combination with monosaccharide composition data, support classification of the exopolysaccharide as a β-(2→6)-linked levan-type fructan with possible β-(2→1) branching and minor terminal glucose residues, which aligns with previously reported native levan-type fructans produced by LAB strains **[14, 61, S6]**.

***UV-Vis spectrum***

The UV–visible spectral profile of the purified levan **(Fig. S3)** exhibited a strong absorption band in the range of 190–220 nm, characteristic of carbohydrate polymers and attributed primarily to *σ→σ** and *n→σ** electronic transitions associated with C–O and C–O–C bonds in the polysaccharide backbone **[S9]**. The previously reported minor absorbance in the 250–280 nm region may reflect trace aromatic impurities; however, its low intensity suggests negligible protein or nucleic acid contamination **[S2]**, and the absence of significant absorbance above 300 nm further supports the purity. Overall, the UV–visible profile is consistent with a carbohydrate-dominated macromolecule lacking substantial chromophoric contaminants. Collectively, the concordant FT-IR, NMR and UV–Vis spectroscopic data confirm that the polymer is a high-purity fructan-type levan predominantly composed of β-D-fructofuranose units, consistent with the exopolysaccharide biosynthesis by *Weissella* species **[S6]**.

**Supplementary Figures**

**Fig. (S1):** Determination of sample size using G-Power software. The Priori test analysis of the two independent groups (vehicle control and untreated EAC positive control) revealed an ~11.527-fold difference in alpha fetoprotein (AFP) levels in tin the untreated EAC group relative to the vehicle group, with a minimum of 2 mice per group required to fulfill an effect size (f) of 11.527 and a study power of 95% (1-β error probe).

**Fig. (S2):** FT-IR spectrum of the purified levan. The broad absorption band at ~3438 cm⁻¹ corresponds to O–H stretching vibrations of hydroxyl groups in the polysaccharide backbone, indicating extensive hydrogen bonding. Bands at 2929 and 2841 cm⁻¹ are attributed to C–H stretching vibrations of aliphatic groups. The band at 1637 cm⁻¹ is primarily assigned to H–O–H bending of bound water molecules. Prominent absorptions at 1142 and ~976 cm⁻¹ correspond to C–O–C glycosidic linkages and furanose ring vibrations characteristic of fructan-type polysaccharides. Weak bands observed in the 1250–1300 cm⁻¹ region and near ~ 555 cm⁻¹ falls within spectral regions of sulfate-related vibrations; however, their low intensity suggest that these bands may arise from trace sulfate residues originating from the fermentation medium.

**Fig. (S3):** ^1H NMR spectrum of the purified levan recorded in DMSO-*d*₆. The spectrum is dominated by signals between 3.6–4.4 ppm corresponding to ring protons of β-D-fructofuranosyl residues. Weak signals in the 4.9–5.4 ppm region are attributed to minor α-D-glucopyranosyl anomeric protons. The spectral profile is consistent with a β-(2→6)-linked fructan backbone with limited branching and minor terminal glucose residues.

**Fig. (S4):** UV–visible absorption spectrum of the purified levan. The spectrum shows strong absorption in the 190–220 nm range, characteristic of polysaccharides due to *σ→σ** and *n→σ** electronic transitions associated with C–O and C–O–C bonds of the carbohydrate backbone. The weak shoulder observed around 260–280 nm might suggest minor contamination with protein or nucleic acid, but the absence of absorbance > 300 nm supports the overall purity of levan preparation.

**Supplementary Tables**

**Supplementary Table 1. Optimized parameters for the LC-ESI-MS/MS analysis of parent (unmetabolized) BMPE and curcumin concentrations in EAC liver homogenates**

| ID | Q1  (*m/z*) | Q3  (*m/z*) | Dwell time (msec) | DP  (V) | EP  (V) | CE  (V) | CXP  (V) |
| --- | --- | --- | --- | --- | --- | --- | --- |
| BMPE_1 | 252.5 | 209.02 | 200 | 60 | 10 | 20 | 10 |
| BMPE_2 | 252.5 | 224.09 | 200 | 60 | 10 | 25 | 10 |
| Curcumin_1 | 369.1 | 177.05 | 200 | 60 | 10 | 40 | 10 |
| Curcumin _2 | 369.1 | 265.11 | 200 | 60 | 10 | 25 | 10 |

**Supplementary Table 2.** **Primer sets used for profiling the inflammatory and metabolic gene expression in EAC livers**

| Gene | Primer sequence | Annealing Temp. | *Primer Efficiency | Ref |
| --- | --- | --- | --- | --- |
| *MyD88* | F: 5’- CCCACAAACAAAGGAACTGG-3’  R: 5’- TCATCTCCTGCACAAACTCG-3’ | 55 °C | 92.14% | **[36]** |
| *NF-κB* | F: 5’- CGGTGAAACTCTGGCTAGACAG-3’  R: 5’- GCTGCCAAAGAAGGACACGACA-3’ | 58 °C | 101.77% | **[14]** |
| *FXR* | F: 5’- CTTGATGTGCTACAAAAGCTGTG-3’  R: 5’- ACTCTCCAAGACATCAGCATCTC-3’ | 56 °C | 98.36% | **[37]** |
| *FGF15* | F: 5’- CCTGCCTGGTGGCTCTGTCTC-3’  R: 5’- ATAATCCGCAACTCCTCCCGCC-3’ | 63 °C | 95.29% | **[38]** |
| *GAPDH* | F: 5’- CATCACTGCCACCCAGAAGACTG-3’  R: 5’- ATGCCAGTGAGCTTCCCGTTCAG-3’ | 58 °C | – | **[14]** |

*Primer efficiency was calculated based on the slope of the standard curve established between Ct and log DNA copy number (amplification factor = 2) using the calculator of Thermo Scientific Web Tools available from <https://www.thermofisher.com/eg/en/home/brands/thermo-scientific/molecular-biology/molecular-biology-learning-center/molecular-biology-resource-library/thermo-scientific-web-tools/qpcr-efficiency-calculator.html>

**Supplementary Table 3. Isobolographic analysis of BMPE-Cur – levan interactions**

| Test | Combination Index (CI) Value | | | | Interaction |
| --- | --- | --- | --- | --- | --- |
|  | **ED_50_** | **ED_75_** | **ED_90_** | **ED_95_** |  |
| Anti-angiogenic activity | 6.32E^-5^ | 8.561E^9^ | 1.04E^29^ | 9.95E^41^ | Strong Synergism |
| Peripheral AFP | 1.44E^-8^ | 3.7E^-17^ | 9.6E^-26^ | 1.4E^-31^ | Strong Synergism |
| Hepatic AFP | 4.59E^-5^ | 1.62E^57^ | 5.7E^118^ | 4.2E^160^ | Strong Synergism |
| Peripheral CEA | 9.60E^-5^ | 9.22E^-6^ | 1.02E^-6^ | 2.28E^-7^ | Strong Synergism |
| Hepatic CEA | 1.33E^-5^ | 1.11E^-5^ | 1.11E^-5^ | 1.11E^-5^ | Strong Synergism |
| Peripheral VEGFR | 4.61E^-4^ | 2.11E^-7^ | 9.6E^-11^ | 5.2E^-13^ | Strong Synergism |
| Hepatic VEGFR | 0.0016 | 6.59E^-6^ | 2.71E^-8^ | 6.5E^-10^ | Strong Synergism |
| Peripheral IL-6 | 0.0039 | 91210 | 2.86E^13^ | 1.98E^19^ | Strong Synergism |
| Hepatic IL-6 | 0.0053 | 1.13E^-4^ | 2.42E^-6^ | 1.77E^-7^ | Strong Synergism |
| Peripheral TNF-α | 0.031 | 0.0297 | 0.0297 | 0.0297 | Strong Synergism |
| Hepatic TNF-α | 8.19E^-4^ | 4.0E^-24^ | 3.3E^-43^ | 3.4E^-56^ | Strong Synergism |
| Peripheral IL-10 | 4.826 | 0.7734 | 0.1239 | 0.0357 | Antagonism |
| Hepatic IL-10 | 3.10E^-4^ | 2.38E^19^ | 1.90E^42^ | 7.16E^57^ | Strong Synergism |
| Peripheral CXCL1 | 3.86E^-6^ | 10.E^-15^ | 2.6E^-23^ | 3.7E^-29^ | Strong Synergism |
| Hepatic CXCL1 | 0.0061 | 6.0E^-14^ | 1.7E^-23^ | 5.6E^-30^ | Strong Synergism |
| Peripheral CD4^+^ | 1.66E^11^ | 7.15E^18^ | 3.08E^26^ | 4.79E^31^ | Antagonism |
| Hepatic CD4^+^ | 4.25E^-5^ | 1.12E^-8^ | 3.0E^-12^ | 1.1E^-14^ | Strong Synergism |
| Peripheral CD8^+^ | 2.99E^-4^ | 3.65E^-9^ | 5.5E^-13^ | 1.4E^-15^ | Strong Synergism |
| Hepatic CD8^+^ | 6.40E^-7^ | 3.8E^-23^ | 2.3E^-39^ | 2.2E^-50^ | Strong Synergism |
| Peripheral CD25^+^ | 0.7335 | 0.0815 | 0.0091 | 0.00203 | Synergism |
| Hepatic CD25^+^ | 0.0026 | 111108 | 4.78E^12^ | 7.44E^17^ | Strong Synergism |
| Hepatic TLR2 | 9.11E^-7^ | 2.4E^-10^ | 6.3E^-14^ | 2.3E^-16^ | Strong Synergism |
| Hepatic *MyD88* | 4.68E^-5^ | 8.40E^17^ | 4.39E^44^ | 6.54E^62^ | Strong Synergism |
| Hepatic *NF-κB* | 6.34E^-4^ | 2.90E^-7^ | 1.3E^-10^ | 7.1E^-13^ | Strong Synergism |
| Hepatic *FXR* | 1.96E^-9^ | 7.3E^-36^ | 2.7E^-62^ | 2.8E^-80^ | Strong Synergism |
| Hepatic *FGF15* | 0.0015 | 3.9E^-12^ | 10.E^-21^ | 1.4E^-26^ | Strong Synergism |
| Hepatic NO | 7.06E^-6^ | 9.25E^50^ | 1.7E^108^ | 1.5E^147^ | Strong Synergism |
| Hepatic MDA | 2.18E^-5^ | 2.16E^-8^ | 1.5E^-10^ | 7.5E^-12^ | Strong Synergism |
| Hepatic GSH | 2.47E^-6^ | 1.3E^-10^ | 6.4E^-15^ | 7.7E^-18^ | Strong Synergism |
| Hepatic catalase | 9.53E^-4^ | 1.3E^-11^ | 1.7E^-19^ | 7.6E^-25^ | Strong Synergism |
| Hepatic SOD | 0.00138 | 3.6E^-12^ | 9.2E^-21^ | 1.3E^-26^ | Strong Synergism |

**Supplementary References**

S1. Xu, R.B., Yang, X., Wang, J., Zhao, H.T., Lu, W.H., Cui, J., Cheng, C.L., Zou, P., Huang, W.W., Wang, P. & Li, W.J. Chemical composition and antioxidant activities of three polysaccharide fractions from pine cones. Int. J. Mol. Sci. **13**, 14262-14277 (2012). <https://doi.org/10.3390/ijms131114262>
S2. Liu, D., Liao, N., Ye, X., Hu, Y., Wu, D., Guo, X., Zhong, J., Wu, J. & Chen, S. Isolation and structural characterization of a novel antioxidant mannoglucan from a marine bubble snail, *Bullacta exarata* (Philippi). Mar. Drugs. **11**, 4464-4477 (2013). <https://doi.org/10.3390/md11114464>
S3. Hong, T., Yin, J.Y., Nie, S.P. & Xie, M.Y. Applications of infrared spectroscopy in polysaccharide structural analysis: Progress, challenge and perspective. Food Chem. X. **12**, 100168 (2021). <https://doi.org/10.1016/j.fochx.2021.100168>
S4. Soto-Vásquez, M.R., Alvarado-García, P.A.A., Youssef, F.S., Ashour, M.L., Bogari, H.A. & Elhady, S.S. FTIR characterization of sulfated polysaccharides obtained from *Macrocystis integrifolia* algae and verification of their antiangiogenic and immunomodulatory potency *in vitro* and *in vivo*. Mar. Drugs. **21**, 36 (2022). <https://doi.org/10.3390/md21010036>
S5. Deveci, E., Tel-Cayan, G., Cayan, F., Yılmaz Altınok, B. & Aktas, S. Characterization of polysaccharide extracts of four edible mushrooms and determination of *in vitro* antioxidant, enzyme inhibition and anticancer activities. ACS Omega. **9**, 25887-25901 (2024).  <https://doi.org/10.1021/acsomega.4c00322>
S6. Kavitake, D., Devi, P.B. & Shetty, P.H. Overview of exopolysaccharides produced by *Weissella* genus - A review. Int. J. Biol. Macromol. **164**, 2964-2973 (2020). <https://doi.org/10.1016/j.ijbiomac.2020.08.185>
S7. Ando, I. Some aspects of the NMR chemical shift/structure correlation in the structural characterization of polymers and biopolymers. Polymer J. **44**, 734-747 (2012). <https://doi.org/10.1038/pj.2012.95>
S8. Ismail, B. & Nampoothiri, K.M. Production, purification and structural characterization of an exopolysaccharide produced by a probiotic *Lactobacillus plantarum* MTCC 9510. Arch. Microbiol. **192**, 1049-1057 (2010). <https://doi.org/10.1007/s00203-010-0636-y>
S9. Xiao, L., Xu, D., Tang, N., Rui, X., Zhang, Q., Chen, X., Dong, M. & Li, W. Biosynthesis of exopolysaccharide and structural characterization by *Lacticaseibacillus paracasei* ZY-1 isolated from Tibetan kefir. Food Chem. Mol. Sci. **3**, 100054 (2021). <https://doi.org/10.1016/j.fochms.2021.100054>
