# Supplementary material for: Microbial levan potentiates hepatic retention and antitumor activity of a PEGylated benzimidazole–curcumin nanocomplex through TLR2–FXR/FGF15-associated immunometabolic remodeling in experimental liver cancer
Source: Sci Rep. 2026 Aug 12;16:25094. doi: 10.1038/s41598-026-64576-8 (PMC13462577; doi:10.1038/s41598-026-64576-8)

Created with SCIEX OS 1.6.2
Printed:11/2/2025 8:04:22 PM

Analyte Name: BMPE_1
Internal Standard: N/A

**Data File**	02112025.wiff	**Result Table**	02112025
**Acquisition Date**	11/2/2025 4:15:55 PM	**Algorithm Used**	AutoPeak
**Acquisition Method**	sMRM_BMPE_Curc_0.8.dam	**Instrument Name**	QTRAP Enabled Triple Quad 5500+
**Project**	N/A	**Processing Method**	02112025.qmethod

Regression Equation: y = 2896.11613 x + 10.46878 (r = 0.99953, r² = 0.99906) (weighting: 1 / x)

**Expected Concentration**	**Number of Values**	**Mean Calculated Concentration (No data for Analyte Unit)**	**% Accuracy**	**Std. Deviation**	**%CV**
1.00	1 of 1	9.752e-1	97.5	N/A	N/A
5.00	1 of 1	4.849e0	97.0	N/A	N/A
25.00	1 of 1	2.665e1	106.6	N/A	N/A
50.00	1 of 1	5.038e1	100.8	N/A	N/A
100.00	1 of 1	9.814e1	98.1	N/A	N/A

Analyte Name: BMPE_1

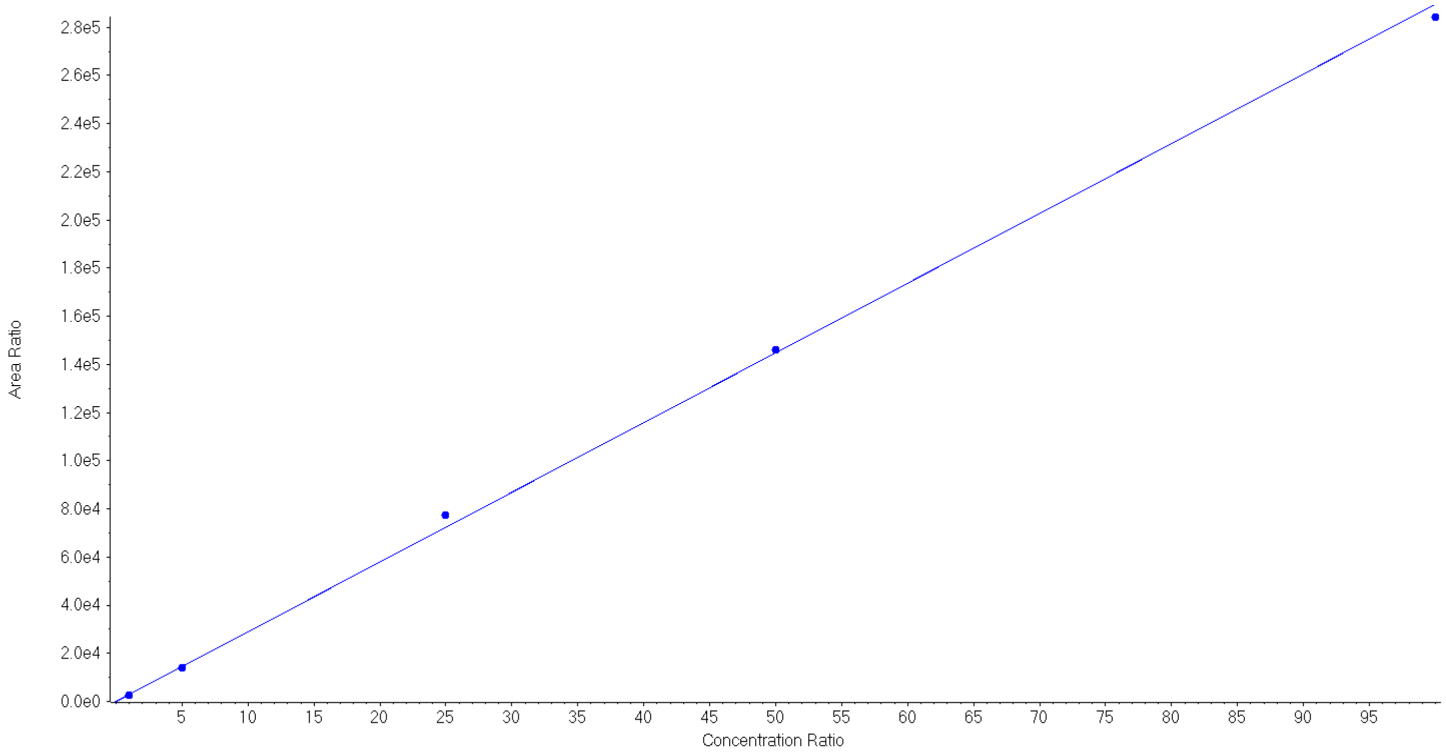

Analyte Name: BMPE_2
Internal Standard: N/A

**Data File**	02112025.wiff	**Result Table**	02112025
**Acquisition Date**	11/2/2025 4:15:55 PM	**Algorithm Used**	AutoPeak
**Acquisition Method**	sMRM_BMPE_Curc_0.8.dam	**Instrument Name**	QTRAP Enabled Triple Quad 5500+
**Project**	N/A	**Processing Method**	02112025.qmethod

Regression Equation: y = 394.09809 x + 358.05704 (r = 0.99805, r² = 0.99611) (weighting: 1 / x)

Expected Concentration	Number of Values	Mean Calculated Concentration (No data for Analyte Unit)	% Accuracy	Std. Deviation	%CV
1.00	0 of 1	N/A	N/A	N/A	N/A
5.00	1 of 1	4.794e0	95.9	N/A	N/A
25.00	1 of 1	2.741e1	109.6	N/A	N/A
50.00	1 of 1	4.669e1	93.4	N/A	N/A
100.00	1 of 1	1.011e2	101.1	N/A	N/A

Analyte Name: BMPE_2

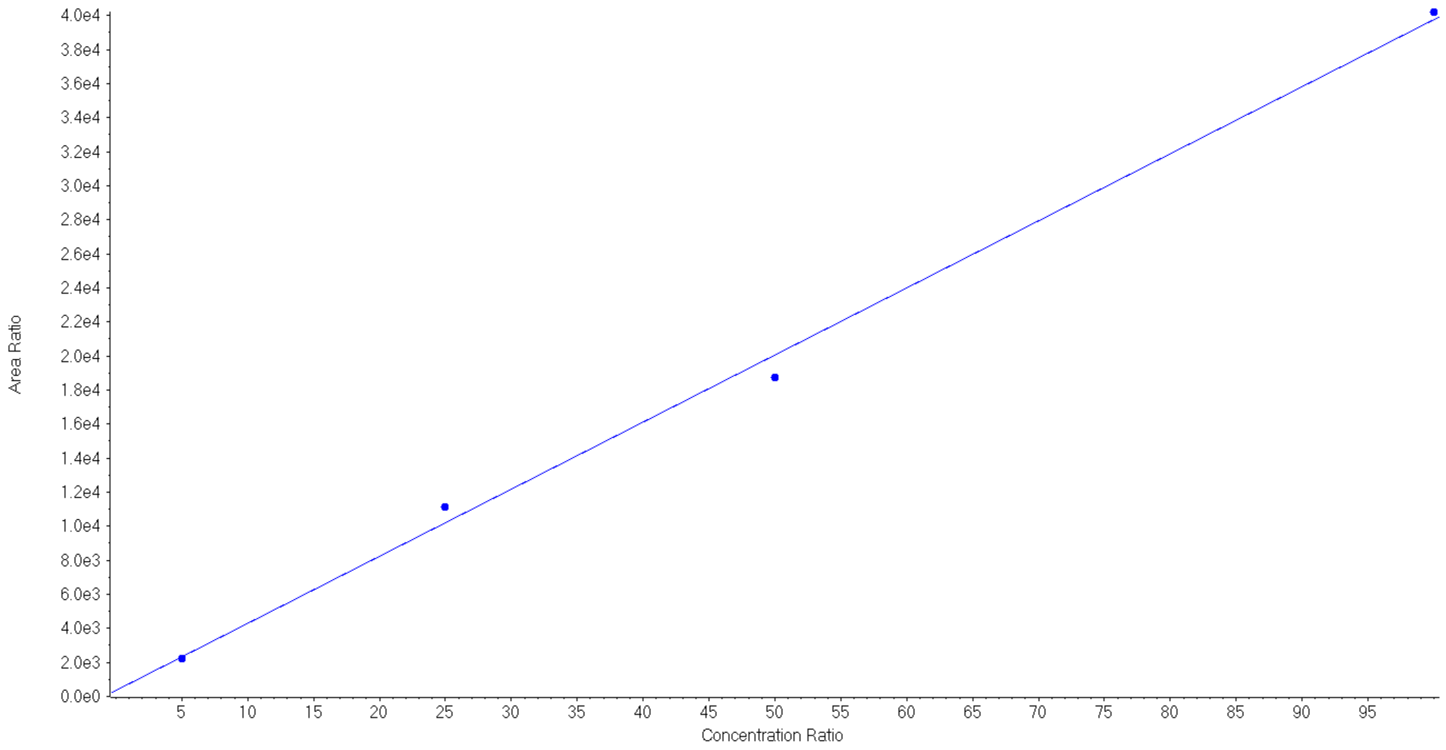

Analyte Name: Curcumin_1
Internal Standard: N/A

**Data File**	02112025.wiff	**Result Table**	02112025
**Acquisition Date**	11/2/2025 4:15:55 PM	**Algorithm Used**	AutoPeak
**Acquisition Method**	sMRM_BMPE_Curc_0.8.dam	**Instrument Name**	QTRAP Enabled Triple Quad 5500+
**Project**	N/A	**Processing Method**	02112025.qmethod

Regression Equation: y = 7469.36173 x + -1341.79393 (r = 0.99909, r^2 = 0.99818) (weighting: 1 / x)

Expected Concentration	Number of Values	Mean Calculated Concentration (No data for Analyte Unit)	% Accuracy	Std. Deviation	%CV
1.00	1 of 1	1.082e0	108.2	N/A	N/A
5.00	1 of 1	4.891e0	97.8	N/A	N/A
25.00	1 of 1	2.372e1	94.9	N/A	N/A
50.00	1 of 1	4.780e1	95.6	N/A	N/A
100.00	1 of 1	1.035e2	103.5	N/A	N/A

Analyte Name: Curcumin_1

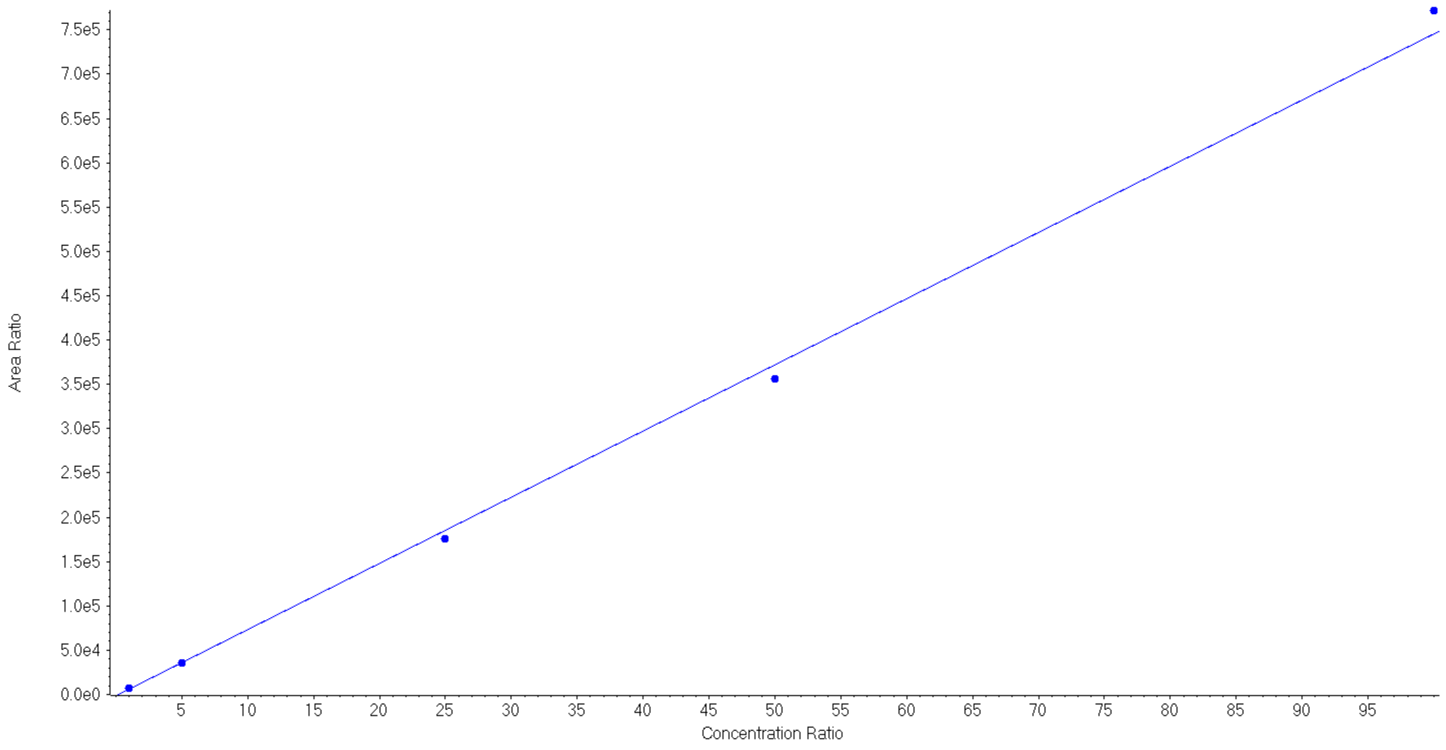

Analyte Name: Curcumin_2
Internal Standard: N/A

**Data File**	02112025.wiff	**Result Table**	02112025
**Acquisition Date**	11/2/2025 4:15:55 PM	**Algorithm Used**	AutoPeak
**Acquisition Method**	sMRM_BMPE_Curc_0.8.dam	**Instrument Name**	QTRAP Enabled Triple Quad 5500+
**Project**	N/A	**Processing Method**	02112025.qmethod

Regression Equation: y = 9737.89729 x + -1771.52541 (r = 0.99921, r^2 = 0.99841) (weighting: 1 / x)

Expected Concentration	Number of Values	Mean Calculated Concentration (No data for Analyte Unit)	% Accuracy	Std. Deviation	%CV
1.00	1 of 1	1.092e0	109.2	N/A	N/A
5.00	1 of 1	4.818e0	96.4	N/A	N/A
25.00	1 of 1	2.376e1	95.0	N/A	N/A
50.00	1 of 1	4.808e1	96.2	N/A	N/A
100.00	1 of 1	1.033e2	103.3	N/A	N/A

Analyte Name: Curcumin_2

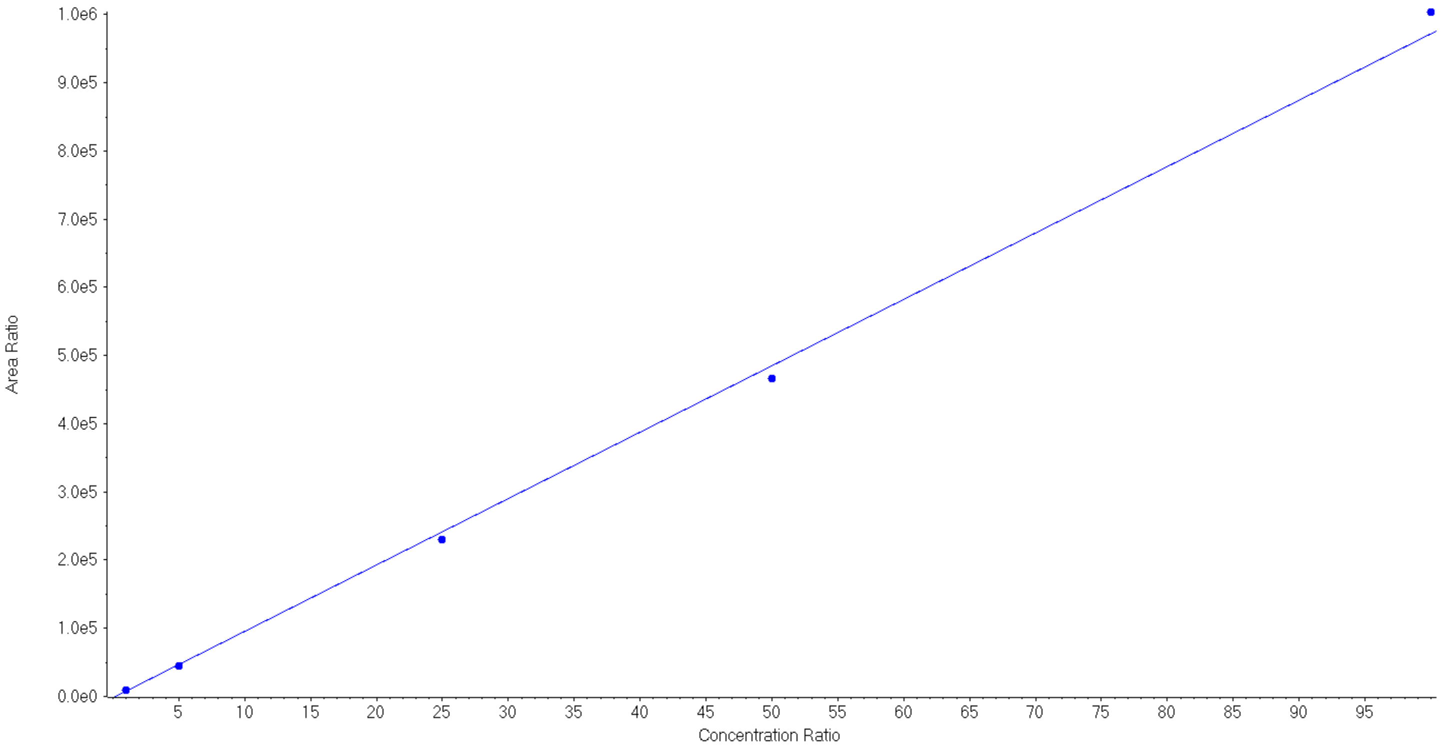

Supplement: Supplementary file 1 [file 41598_2026_64576_MOESM1_ESM.pdf]
